# Report of the fourth conference on next-generation sequencing (NGS) for adventitious virus detection in biologics for humans and animals: Validation and implementation of NGS

**DOI:** 10.1016/j.biologicals.2025.101859

**Published:** 2025-11

**Authors:** Arifa S. Khan, Laurent Mallet, Johannes Blümel, Noémie Deneyer, Sigrid De C.J. Keersmaecker, Blandine de Saint-Vis, Ivana Knezevic, Carine Logvinoff, Marie Murphy, Siemon H.S. Ng, Yoji Sato, Michael Wall, Ana Goios, Pieter Neels

**Affiliations:** a Division of Viral Products, Office of Vaccines Research and Review, Center for Biologics Evaluation and Research, U.S. Food and Drug Administration, Silver Spring, MD, USA; b European Directorate for the Quality of Medicines & HealthCare, Strasbourg, France; c Paul-Ehrlich-Institut, Langen, Germany; d GSK, Wavre, Avenue Fleming 20, 1300, Wavre, Belgium; e Sciensano, Bruxelles, Belgium; f Boehringer Ingelheim Animal Health, Saint-Priest, France; g Department of Health Products Policy and Standards, World Health Organization, Geneva, Switzerland; h Sanofi, Marcy L'Etoile, France; i Eli Lilly & Company, Cork, Ireland; j Notch Therapeutics, Toronto, ON, Canada; k National Institute of Health Sciences, Kawasaki, Kanagawa, Japan; l Health Canada, Ottawa, ON, Canada; m P95 Clinical and Epidemiology Services, Leuven, Belgium; n International Alliance for Biological Standardization, Geneva, Switzerland

**Keywords:** Vaccines, Biologics, Medical products, Cell substrates, Adventitious viruses, Next-generation sequencing, High-throughput sequencing, *In vivo* assays, Bioinformatics

## Abstract

This report is a summary of the 4th Conference on NGS for Adventitious Virus Detection, which took place on December 4–5, 2024, in Frankfurt, Germany, and was sponsored by the International Alliance for Biological Standardization (IABS), and co-chaired by the U.S. Food and Drug Administration (FDA) and the European Directorate for the Quality of Medicines & HealthCare (EDQM). The increased interest in using NGS for adventitious virus detection follows its recent introduction in the ICH Q5A (R2) guideline and the new EDQM/European Pharmacopoeia general chapter 2.6.41. Key conference objectives included evaluating NGS validation and implementation, addressing regional challenges, and discussing regulatory acceptance as an alternative method to the conventional assays. The conference fostered networking between early and established NGS users and emphasized the Advanced Virus Detection Technologies Working Group as a key learning hub for NGS applications. Discussions focused on method validation requirements and the need for defining a specific limit of detection. Participants shared updates on scientific developments and regulatory submissions. A general consensus was reached on the readiness of NGS to replace the *in vivo* adventitious virus detection assays and PCR assays, and to supplement or replace the *in vitro* cell-based assays, based on a suitable validation package.

## Highlights

• This report summarized the 4th Conference on NGS for Adventitious Virus Detection in Biologics, wheld on December 4–5, 2024.
• Interest in using NGS for adventitious virus detection has increased since its introduction in the ICH Q5A (R2) guideline.
• NGS validation and implementation, regional challenges, and regulatory acceptance of using NGS was discussed.
• Discussions focused on method validation requirements and the need for defining a specific limit of detection.
• Consensus was reached on the readiness of NGS to replace the *in vivo* adventitious virus detection assays and PCR assays.

**Table:** Abbreviations

AI	Artificial intelligence
ATCC	American Type Culture Collection
ATMP	Advanced Therapy Medicinal Product
AV	Adventitious virus
AVDT(I/W)G	Advanced Virus Detection Technologies (Interest/Working) Group
AZ	AstraZeneca
BLAST	Basic Local Alignment Search Tool
CAACB	Consortium on Adventitious Agent Contamination in Biomanufacturing
CAR-T	Chimeric antigen receptor T
CBER	FDA Center for Biologics Evaluation and Research
CC	Collaborating Centre (WHO's)
CDER	FDA Center for Drugs Evaluation and Research
CHO	Chinese hamster ovary
CPE	Cytopathic effect
CQA	Critical quality attribute
CRO	Contract research organization
ddPCR	Digital droplet PCR
DNA	Deoxyribonucleic acid
EBV	Epstein–Barr virus
ECBS	Expert Committee on Biological Standardization
EDA	Egyptian Drug Authority
EDQM	European Directorate for the Quality of Medicines & HealthCare
EFPIA	European Federation of Pharmaceutical Industries and Associations
EMA	European Medicines Agency
ERCC	External RNA Controls Consortium
EU	European Union
FDA	U.S. Food and Drug Administration
FeLV	Feline leukemia virus
GBT	Global Benchmarking Tool
GMP	Good manufacturing practice
GOI	Gene of interest
H(B/C/E)V	Hepatitis B/C/E virus
HA	Hemagglutination
HAD	Hemadsorption
HHV	Human herpesvirus
HIV	Human immunodeficiency virus
HIVE	High-performance Integrated Virtual Environment
HTS	High-throughput sequencing
IABS	International Alliance for Biological Standardization
ICH	International Council for the Harmonization of Technical Requirements for Pharmaceuticals for Human Use
ITR	Inverted terminal repeat
LAIV	Live attenuated influenza vaccine
LOD	Limit of detection
MAG	Metagenome-assembled genome
MAP/RAP/HAP	Mouse/Rat/Hamster Antibody Production Tests
MHRA	Medicines and Healthcare products Regulatory Agency
MIT	Massachusetts Institute of Technology
ML	Machine learning
mNGS	Metagenomics NGS
MVM	Minute Virus of Mice
NCBI	National Center for Biotechnology Information
NGS	Next-generation sequencing
NIBSC	National Institute for Biological Standards and Control
NIH	U.S. National Institutes of Health
NIIMBL	National Institute for Innovation in Manufacturing Biopharmaceuticals
NR/NT	non-redundant nucleotide collection
NRA	National Regulatory Authority
ONT	Oxford Nanopore Technology
ORF	Open Reading Frame
OVRR	Office of Vaccines Research and Review
PBMC	Peripheral blood mononuclear cell
PCR	Polymerase Chain Reaction
PCV	Porcine Circovirus
PDA	Parenteral Drug Association
PEI	Paul-Ehrlich-Institut
Ph. Eur.	European Pharmacopoeia
QC	Quality control
rAAV	Recombinant adeno-associated virus
RefSeq	NCBI Reference Sequence Database
REO	Reovirus
RNA	Ribonucleic Acid
RSV	Respiratory syncytial virus
RT	Reverse transcriptase
RVDB	Reference Virus Database
SOP	Standard operating procedure
TAT	Turn-around time
TRS	Technical Report Series
VGC	Viral genome copies
VRBPAC	Vaccines and Related Biological Products Advisory Committee
WHO	World Health Organization

## 1 Introduction

Next-generation sequencing (NGS) or high-throughput sequencing (HTS) – terms which are used interchangeably in this report – has been recognized as a powerful technology that can overcome the limitations of conventional *in vivo* and *in vitro* testing methods for adventitious virus (AV) detection in biologics. The 4th Conference on NGS for Adventitious Virus Detection in Biologics for Humans and Animals was held on 4–5 December 2024 in Frankfurt, Germany, sponsored by the International Alliance for Biological Standardization (IABS) and co-chaired by **Arifa S. Khan** from the U.S. Food and Drug Administration (FDA) and **Laurent Mallet** from the European Directorate for the Quality of Medicines & HealthCare (EDQM). The meeting focused on the applications of NGS for AV testing of different biological materials involved at different stages of production of biologics. The main aims were to determine the status of NGS validation and implementation and to identify the challenges and gaps remaining for establishing or broadening the use of NGS across different geographic regions. Additionally, an overall goal was to determine the acceptance by regulatory authorities for using NGS as an alternative method for replacing or supplementing the conventional AV detection assays. It was noted that the interest in using NGS for AV detection has increased since its recent introduction in the ICH Q5A (R2) guideline (November 2023) [1] and the drafting of the EDQM/European Pharmacopoeia (Ph.Eur.) general chapter 2.6.41 on “High-throughput sequencing for the detection of viral extraneous agent” (to be published in 2025).

The conference was preceded by two technical training sessions that were co-chaired by Arifa S. Khan and Laurent Mallet: a virtual NGS webinar [2] held on September 24–25, 2024 and a hybrid NGS training workshop held on December 3, 2024. These sessions were aimed at providing background information on NGS technologies to new and early users to facilitate the understanding of the advanced technical and bioinformatics presentations at the 4th NGS conference.

The **NGS webinar** introduced NGS technologies and their applications in the viral safety testing package of biological products. It specifically covered a review of the sources of introducing adventitious viral contamination in the biomanufacturing process, reports of cases of contamination with adventitious viruses, considerations for using NGS as an alternative method for the current AV detection assays, and an overview of the different steps in the NGS workflow. The webinar was intended for manufacturers and regulators who want to learn about NGS technologies and their applications for adventitious virus detection in biological products. The presentations and discussions focused on how NGS could be used to enhance the viral safety of biologics [2]. The webinar also provided an overview of the Advanced Virus Detection Technologies Working Group (AVDTWG) activities and an update on the regulatory landscape. There were 297 participants in the webinar.

The pre-conference, **NGS training workshop** was a hybrid event to include participation from diverse geographic regions and provide opportunities to establish networking with NGS experts. The talks covered a general overview of how NGS was introduced for AV detection in biologics and how it can address the limitations of the current *in vitro* and *in vivo* AV detection assays; the ongoing efforts and contributions of the AVDTWG; technical presentations on three NGS technologies by their suppliers; a detailed description of the different steps in the NGS workflow [sample and library preparation, bioinformatics tools, strategies and analysis of NGS data using non-targeted and targeted approaches and use of databases including the Reference Virus Database (RVDB)]; and follow-up investigations needed for confirming a true viral signal. The workshop sessions concluded with a panel discussion. The speakers and panelists were from regulatory agencies, human and animal vaccine industry representatives, gene therapy industry representative and suppliers of NGS technologies.

The workshop was followed by the **AVDTWG session**, which was open to all onsite conference attendees. This session highlighted the working groups’ activities and achievements and described the various spiking studies for evaluating NGS short-read and long-read technologies in various matrices relevant to different biological materials tested for AV in manufacturing. It also provided an opportunity to meet and greet the working group members, and extended opportunities for networking between new, early, and advanced NGS users for knowledge exchange and collaborations.

The **4**th **NGS Conference**, which is detailed in this report, brought together scientists representing industry, academia, contract research organizations (CROs), and regulatory and health agencies, to discuss the status of NGS validation and implementation for AV testing in biologicals. The conference was officially opened by **Rick Hill**, IABS President, who highlighted the progress accomplished so far in bringing NGS technology from concept to implementation in improving the safety of biologicals.

One hundred seventy-five participants attended the workshop and the conference, virtually or in person, from various geographical regions in Europe, North America, Asia, Africa, and South America.

### 1.1 Summary of previous meetings and goal of the 4th meeting

**Arifa Khan** presented a summary of the previous IABS NGS meetings. It was reminded that the consideration for using NGS for AV detection in biologics was accelerated by industry and regulatory agencies by the unexpected discovery of porcine circovirus type 1 (PCV1) in a licensed rotavirus vaccine using NGS and virus microarrays in 2010 [3]. The FDA and other organizations followed this topic by dedicating efforts to discussing and evaluating the applications of NGS for AV detection in biologics by forming a small Advanced Virus Detection Technologies User Group in 2012, which then became an Interest Group [4], and in 2022 was designated as the Advanced Virus Detection Technologies Working Group (AVDTWG).

The first IABS NGS Conference, which was held in 2017 in Rockville, Maryland, U.S.A. [5], identified the challenges of using NGS, which included: need for developing sample preparation approaches, reference standard reagents, well-annotated databases, large data storage and transfer capacity, and a clear and simple strategy for follow-up of NGS hits resulting from bioinformatics data analysis. Additionally, the need for collaboration involving experts in virology, bioinformatics, and computation, as well as harmonization of international regulations on this topic, was recognized.

The second IABS NGS Conference was held in 2019 in Ghent, Belgium [6]. It was recognized that NGS could be as sensitive for virus detection as PCR assays and used as a valuable investigation technique. It was noted that validation data were needed to support its use as a replacement assay for the conventional *in vivo* testing for AVs, and additional data needed to be generated to support replacing the *in vitro* cell culture assays.

The third IABS NGS Conference was held in 2022 in Rockville, Maryland, U.S.A. [7]. The implementation of NGS by various companies was presented, which included method validation, as well as increasing submissions to some regulatory agencies, such as U.S. FDA and EMA. This was facilitated by the availability of: 1) the five model virus stocks developed by the U.S. FDA Center for Biologics Evaluation and Research (CBER) and adopted in 2020 as the WHO International Reference Reagents [8,9], which are currently designated as CBER NGS Virus Reagents following their replacement by the First WHO International Reference Panel in 2024 [10]; and 2) the Reference Virus Database (RVDB) [11]. Additionally, new guidelines under development were presented, including the ICH Q5A(R2) [1], and the European Pharmacopoeia (Ph. Eur.) draft chapter 2.6.41 describing the NGS technology and validation approaches [12].

**Laurent Mallet** presented the goals of the 4th IABS NGS Conference, reported here. The main aim was to develop a scientific consensus regarding the implementation of NGS for the detection of AV. It was deemed important to share the latest scientific data regarding NGS application and implementation for the detection of AVs for safety evaluation of biologics and the latest updates regarding the introduction of NGS in regulatory documents. The goal was also to discuss the status of NGS in regulatory submissions and industry applications, and the expectations for the validation of NGS for viral safety of biologics.

## 2 Session 1: current perspectives on using NGS for adventitious virus testing

This session was moderated by the meeting co-chairs Laurent Mallet (EDQM, France) and Arifa Khan (FDA-CBER, U.S.A.).

### 2.1 International council for harmonization (ICH)

**Johannes Blümel** (Paul-Ehrlich-Institut [PEI], Germany) presented on the introduction of NGS for AV detection in the revised ICH Q5A guideline. Originally established in 1997, the ICH Q5A's Revision 2 (R2) was adopted on November 1, 2023 [1]. It addresses new product types that are amenable to viral clearance (including genetically engineered viral vectors and viral vector-derived products), new test methods and new analytical technologies (such as NGS), manufacturing and biotechnology advancements, and alternative virus clearance validation strategies (including using prior knowledge). In section 3.1.1 outlining testing of the master cell bank, the revision states that “Testing for adventitious viruses should include both broad and specific virus detection assays,” and the “introduction of new methodologies such as NGS for detecting a broad range of adventitious viruses is encouraged.” Regarding this latter topic, the philosophy of the ICH guideline is to use both broad and specific virus detection assays for thorough testing of the cell bank. Traditionally, these have been *in vivo* assays and *in vitro* cell culture assays. However, these assays have limitations (Table 1).

**Table 1:** Advantages and limitations of conventional virus testing assays.

	*In vivo* assays	*In vitro* assays
**Advantages**	• Detection of a range of viruses (may include some unexpected/unknown) and non-cell culture adapted viruses	• Detection of a broad range of viruses (may include some unexpected/unknown) • Sensitive for cell culture adapted virus strains (high volume can be inoculated on indicator cells)

**Limitations**	• Death end-point can be due to multiple causes: injection trauma, bacterial contamination, cannibalization of suckling mice • Toxicity of media components • Active substance (live virus) can interfere with virus detection (neutralization with animal sera required) • Limited sensitivity, except for some specific viruses • Limited specificity • High number of animals used to get valid results	• Specific for a range of viruses based on target cell lines used • Not all viruses induce cytopathic effects or haemadsorption/haemagglutination • Time and labor consuming • Requires correct transport of test items (cooling) • Various factors can interfere with virus detection: o Contamination of indicator cell culture o Components of test matrix o Active substance (live virus) • Problems with neutralization/animal-derived serum

In section 3.2.2, the revised guideline revision encourages the introduction of molecular methods, such as NGS, to replace or supplement the *in vitro* assays: “This can address some of these limitations of the *in vitro* cell culture infectivity assay (e.g., susceptibility of cell lines to infection) and specific limitations of the production system (e.g., test article mediated interference or toxicity).” Furthermore, section 3.2.3 states that NGS can replace the *in vivo* assays thus aligning with the global initiative to replace, reduce, and refine the use of animals in research.

The ICH Q5A (R2) guideline also provides basic principles to evaluate the performance of the different steps in the NGS workflow. Qualification and validation of NGS should use suitable reference materials, such as a panel of viruses with distinct physical, chemical, and genomic characteristics and a comprehensive viral database. Other types of reference materials may be used to evaluate the specific technical and bioinformatic steps. NGS is a limit test, and as such, the performance characteristics (sensitivity/limit of detection [LOD] and specificity/breadth of detection) for validation/qualification should consider the relevant principles of ICH Q2 [13]. It is important to note the distinction between validation and qualification: validation is a verification that the method works, and it requires predefined performance criteria; qualification evaluates the performance of the method's characteristics. The need for performing validation or qualification depends on the intended use: validation is required to replace a previous method, while qualification is enough to supplement existing methods. The ICH Q5A(R2) guideline further states that non-targeted NGS can be used without the need for a head-to-head comparison to replace *in vivo* testing and supplement or replace the *in vitro* cell culture assays for the detection of known and unknown or unexpected viruses.

Concerning the selection of model viruses, the ICH guideline does not specify a panel of viruses for validation. The 1st WHO International Reference Panel for NGS is recommended in the Ph. Eur. 2.6.41 based on its careful selection to represent a range of virus physical and biochemical properties, but certain applications may justify its replacement by other viruses [12]. The NGS method should be capable of detecting viruses with a sufficiently high sensitivity. In this context, it may be useful to compare the sensitivity of the NGS method with published literature or previously obtained data on the sensitivity of *in vivo* and *in vitro* AV testing. However, “non-inferiority” of sensitivity may not be necessary for each case and virus. For replacing or supplementing the *in vitro* cell culture assays, a large number of model viruses should be included in spiking studies for NGS validation, even though not all need to be included in each matrix-specific validation. Matrix-specific validation using the WHO reference panel (or equivalent) is expected.

NGS can be sufficiently sensitive to justify replacing *in vitro* testing. However, the sensitivity of virus detection may be affected by the stage of testing during the manufacturing and the test material: for example, in the case of unprocessed bulk testing if contamination happened at a late stage of production the contaminating virus would have little time to replicate, compared to cell bank testing, where the virus might have amplified over several passages. Sensitivity requirements also depend on whether the products will undergo viral clearance, such as in the case of monoclonal antibodies, or not, as is the general case of vaccines and viral vectors. A more cautious strategy is advised when replacing, rather than when supplementing the existing methods.

### 2.2 European Pharmacopoeia (Ph. Eur)

**Laurent Mallet** and **Gwenaël Cirefice** (EDQM, France) presented on “EDQM/Ph. Eur. perspectives on NGS/HTS”.

The EDQM is a directorate of the Council of Europe, founded in 1949 to contribute to public health and access to good quality medicines and healthcare in Europe. EDQM is responsible for the European Pharmacopoeia (Ph. Eur.) [14], which aims to provide quality standards for all medicinal products and their components. Ph. Eur. standards are binding in the 39 Member States, and they are used as a reference worldwide.

Since 2017, Ph. Eur. Chapters 5.2.3 on testing of cell substrates [15] and 2.6.16 on extraneous agent testing of viral seed lots/harvests [16] have foreseen the use of broad molecular methods (such as NGS) to replace the *in vivo* tests and to replace or supplement the *in vitro* cell culture tests for detection of AVs. Chapter 5.2.14 includes dedicated considerations for the substitution of the *in vivo* methods by broad molecular methods (such as NGS) [17]. Although they are mentioned in the Ph. Eur., NGS methods are not described, and the guidance for their validation is not provided. In the context of the ICH Q5A (R2) and of the availability of the 1st WHO International Reference Panel for NGS AV detection, a new Ph. Eur. chapter on NGS/HTS has been elaborated. This document is entitled “HTS for the detection of viral extraneous agents (chapter 2.6.41)” [12], and it includes a description of the technology and workflow, and guidelines for their validation. This chapter was developed by the HTS Working Party, composed of an international group of regulators, national control labs, and industry, from Europe, the U.S., and Canada. A draft version underwent public consultation, and the stakeholder comments were examined by the group of experts.

The draft chapter 2.6.41 includes four parts [12]. Part 1 introduces the scope of the chapter. To ensure product quality and safety, a comprehensive strategy is established following the viral safety risk assessment principles detailed in Ph. Eur, chapter 5.1.7 [18]. HTS can be introduced in the viral extraneous agent testing package to fill gaps identified through the risk assessment, to replace *in vivo* tests and nucleic acid amplification techniques, or to replace or supplement *in vitro* methods using cell cultures. HTS is especially useful for testing of new cell lines to detect known and unknown viruses, but also of virus seeds and harvests, particularly when there is interference in conventional tests. Different HTS technologies exist, including short-read or long-read sequencing technologies with different read lengths and throughput (number of reads generated). The design of the method may allow the detection of a broad spectrum of viral extraneous agents (known and unknown viruses), with a non-targeted approach using a comprehensive database with viral sequence diversity, or the detection of a range of known viruses (or unknown viruses related to known viruses), with a targeted approach using selective capture or amplification of viral sequences.

Part 2 describes the methods, with the different steps of the workflow: sample pre-treatment, extraction of nucleic acids, post-nucleic acid extraction, treatment, library preparation, sequencing, bioinformatics analysis, scientific evaluation of the results, and follow-up investigation. The selection of the most appropriate HTS approach should consider the potential contaminating viruses and the sample type to be analyzed. Consideration should also be given to the detection of cell-associated and cell-free viruses. Different approaches exist for sample preparation, depending on the test material, for the detection of all viral nucleic acids (genomics), viral RNAs (transcriptomics), or encapsidated viral genomes (viromics). The workflow should have sufficient sensitivity in the relevant sample matrices and for the potential level of contamination at the manufacturing stage. The chapter provides an illustrative figure with examples of the HTS workflow and optional steps.

Sample pre-treatment may increase the sensitivity of HTS by concentrating viral particles or reducing the background cellular nucleic acids. The selection of the most appropriate sequencing technology and platform depends on the intended use and should consider the sample type (e.g., high or low-level of contamination expected), required sequencing depth and coverage, sequencing accuracy, and read length. In the context of virus detection, generating more reads in a sequencing run may increase the probability of detecting the presence of extraneous viruses. An appropriate internal control should be added to the sample prior to loading the sample into the sequencer.

The bioinformatics analysis for virus detection involves building a pipeline that generally includes initial processing of the input raw reads to obtain quality reads. In some cases, this may be followed by *de novo* assembly to generate contigs from overlapping reads, prior to mapping or aligning the reads/contigs using a reference virus or a database of viral sequences. A figure on the bioinformatic analysis is provided in the chapter.

The validity of the HTS run should be based on the recovery of one or more internal controls or on expected results from a control sample run in parallel. The interpretation of the results obtained by the bioinformatics analysis should consider predefined criteria for distinguishing true positives and false positive viral signals. Positive results should be reported at the viral species level. In addition, non-viral and background sequences can occur and should also be documented. Appropriate controls should be in place to capture any cross-contamination from the facilities or instruments. A laboratory-based follow-up is needed to assess whether the viral sequence is associated with an infectious virus when a positive result has been identified. A table with questions to be addressed and example approaches for the investigation is provided in this section.

Controls must be in place for the whole workflow in the routine assay. To ensure that the test's performance is adequate, an external or internal positive control is included in each HTS test run, and other controls may be included for the library preparation and sequencing steps.

Part 3 provides guidelines for HTS method validation. The validation of an HTS method for the detection of extraneous viruses must demonstrate that the method is suitable for the intended purpose, based on the sample type (e.g., cell culture/cell bank, virus seed, harvest) and testing approach. HTS is principally used as a qualitative limit test. Thus, specificity (including breadth of virus detection) and LOD are the parameters to be assessed. HTS requires an end-to-end assay validation and may include subdivision of the workflow into modules corresponding to sample and library preparation, the sequencing instrument, and bioinformatics analysis. This modular validation approach provides more flexibility, potentially reducing the effort required for re-validation when updating individual modules.

The spiking material used for validation of the HTS method must be relevant to the intended purpose and the chosen detection strategy. The use of spiked model viruses is relevant for the viromics and genomics approaches, whereas the use of virus-infected cells is more appropriate for the transcriptomics approach. For genomics and viromics approaches in testing cell substrates/cell banks, virus seeds, and harvests, the reference standards should be viruses representing viral diversity in terms of structure (enveloped, non-enveloped), nucleic acid type (RNA or DNA genome), single-vs double-stranded. Such reference model virus stocks should be characterized for genome copy number, infectious titer, viral genome sequence(s), including any variants, and any additional expected background signals. The *First WHO International Reference Panel for Adventitious Virus Detection in Biological Products by HTS* provides diverse virus families and is recommended as the minimum panel for model viruses for validation.

The LOD is defined as the sensitivity at which viral contaminants would be detectable by the method. The sample should be spiked with a known amount of material to mimic different levels of potential virus contamination and added before any treatment. A minimum of three independent replicates of the end-to-end method is required to demonstrate an appropriate assay LOD unless otherwise justified. The LOD should be reported at the level where all spiked-in viruses are detectable in all replicates to indicate the worst-case LOD. It should be reported as genome copies per milliliter for harvests or virus seeds, as genome copies per cell for the genomics approach, or by the number of transcripts per cell or ratio of infected to non-infected cells for the transcriptomics approach. The specificity is reported to demonstrate the breadth of detection for different types of viruses, as well as the correctness of the identification. The identity of the spiked viruses should be as expected. No false positive viral signal should be detected to confirm specificity. A re-validation may be necessary when part of the method is modified. This can be facilitated by a modular approach.

For product-specific validation, the matrix used during the generic validation needs to be compared with the product-specific matrix to evaluate the potential impact on the LOD and specificity. At least one end-to-end validation run is required to verify that changes to the matrix do not impact the LOD or specificity.

Part 4 addresses targeted HTS, a viral detection method using HTS in which nucleic acids of known and closely related viruses are enriched prior to sequencing. The use of targeted HTS should be based on a risk evaluation as the enrichment techniques rely on the availability of known virus sequences. In addition, the design of the enrichment oligos or probes should ensure that a broad specificity is maintained. The bioinformatics analysis can be modified for targeted analysis using specific virus sequences as reference genomes. Validation of a targeted HTS assay should demonstrate the sensitivity for viral detection as described for the non-targeted HTS approach but using relevant viruses as the spiking material (characterized as for non-targeted HTS approach validation).

This chapter has been revised to incorporate stakeholder comments and is expected to be published in 2025. Other chapters, including 5.2.14 [17], will also be updated to align with this new chapter 2.6.41 [12] and the ICH Q5A (R2) [1].

### 2.3 U S. Food and Drug Administration (FDA)

**Arifa Khan** (FDA-CBER, U.S.A.) presented on “NGS for Adventitious Virus Testing of Biological Products: FDA's Efforts, Experience, and Perspective” and the NGS journey in the Office of Vaccines Research and Review (OVRR).

Interest in NGS for AV detection started early in OVRR. In 1997, in response to the influenza pandemic, there was a plethora of novel cell substrates and the introduction of new manufacturing platforms for vaccine development. Between 1998 and 2012, discussions in FDA's Vaccines and Related Biological Products Advisory Committee (VRBPAC) identified the need for assays that can broadly detect both known and unknown viruses to address the challenges of using novel cell substrates. This triggered research efforts within OVRR to evaluate advanced nucleic acid-based technologies available at the time (e.g., microarrays and long-range PCR mass spectrometry [19,20]) resulting in focused efforts toward NGS. The COVID-19 pandemic raised a more urgent need for industry to use NGS for large, rapid vaccine development by replacing the routine conventional assays for AV testing of biologics [21]. OVRR started receiving submissions using NGS for characterization of novel cell substrates as early as 2009 and, more recently NGS applications have progressed for applying genomics, transcriptomics, and viromics approaches to replace or supplement one or even all of the AV detection assays. These include the currently recommended *in vivo* and *in vitro* assays [21], and additional assays that may be recommended by OVRR on a case-by-case basis, including extended or broader PCR-based assays, expanded cell culture assays adding more target cell lines, and chemical induction assays for detection of latent (endogenous or episomal) viruses.

NGS can be used to address the limitations of the conventional assays presented in Table 1, and ensure broad detection of AVs in live viral vaccines, which is important since the manufacturing of such products cannot include rigorous virus inactivation and removal steps due to the need to retain vaccine potency. Some purification steps may be used for some of the new vaccine platforms (viral-like particles [VLPs], or nanoparticle vaccines), but these cannot achieve the high virus clearance level as for biotherapeutics. In some cases, the vaccine particles themselves can be close to the size of some of the model viruses (e.g., MVM), which brings a challenge to demonstrate the effectiveness of viral clearance by the manufacturing process.

Two specific cases highlight the potential of NGS for known and novel virus detection in biologics: an unexpected finding of a known porcine circovirus type 1 (PCV1) in a licensed rotavirus vaccine [3] and the discovery of a novel rhabdovirus in the Sf9 cell line used for baculovirus-expressed products [22]. In both cases, extensive testing had been done to demonstrate the absence of AVs with the conventional assays recommended at the time. These cases triggered further efforts from the FDA for investigating use of NGS for AV detection in biologics. The FDA and CBER established genomics working groups to create a research regulatory infrastructure to support policy development and decision-making related to applications of NGS. In-house laboratory and bioinformatics expertise for evaluating performance of NGS were strengthened.

Additionally, the PDA-AVDTWG was established to address the challenges for standardization and implementation of AV detection in biologics [4]. This working group started in 2012 as a small User Group, which expanded to an Interest Group in 2014, and to a Working Group in 2022. Its mission is to advance next-generation viral risk evaluation by providing an informal scientific forum for discussions and scientific collaborations, with an ongoing focus on NGS. The group is co-chaired by Arifa Khan (U.S.A.), Siemon Ng (Canada), Ken Kono (Japan) and Noémie Deneyer (Belgium), and it currently has more than 240 participants from more than 60 organizations, including different regulatory agencies, industry and service providers, and academics. General meetings are held online every two months. Subgroups were formed to address the different challenges focused on: sample selection, preparation, and processing, and virus standards/reference materials (subgroup AB); complete and correctly annotated virus reference database development (subgroup C) and bioinformatic pipelines and follow-up strategies to confirm the identity of a hit (subgroup DE).

Subgroup AB has facilitated the organization of several collaborative spiking studies to evaluate the breadth and sensitivity of virus detection in different matrices, using short-read and long-read NGS. The goals of these studies are to evaluate sample preparation approaches, reference standards and bioinformatics tools and to compare and optimize NGS protocols.

CBER/OVRR has established efforts toward NGS implementation. These include collaborative efforts to determine capabilities of NGS platforms for virus detection such as: the development of the WHO International Reference Reagents with 5 viruses (currently designated as CBER NGS Virus Reagents and available for NGS development and advancement (NIAID BEI cat. no. NR-59622) [8,9]; the development of the First WHO International Reference Panel for AV Detection in Biological Products with 7 viruses, and is available for NGS qualification and validation studies (NIAID BEI cat. no. NR-59630) [10]; and the generation of a comprehensive Reference Virus Database (RVDB) with ongoing consultation of the AVDTWG, and freely available for download at https://rvdb.dbi.udel.edu [11]. The work on the RVDB is further detailed in Pei-Ju Chin's presentation (Section 3.5).

The WHO International Reference Panel includes seven viruses representing different virus families, selected based on various properties in terms of the particle size, enveloped or not, genome topology (double-stranded DNA linear, single-stranded RNA dimeric, and segmented), genome size, and physical-chemical resistance, to help evaluate the extraction process itself. The panel includes the five viruses available in the CBER NGS Virus Reagents [8] — Epstein–Barr virus (EBV), feline leukemia virus (FeLV), respiratory syncytial virus (RSV), mammalian orthoreovirus (REO), and PCV1 — plus the human coronavirus OC43 and the Minute Virus of Mice (MVM). Viruses in both the Virus Reagents and the Reference Panel were well-characterized for infectious titer particles, reference virus genome copy number, AV analysis by NGS, host cell DNA copy number, reference virus genome sequences, genome sequence variant analysis, and other parameters. Due to the production cell lines, the EBV stock contains the squirrel monkey retrovirus, and the PCV1 stock contains the porcine endogenous retrovirus. Long-term stability studies are continuing, and each virus is individually vialed to allow freedom for custom mixing as needed by the user.

Efforts for NGS implementation are ongoing through collaborative studies to evaluate the sensitivity of virus detection in complex matrices and develop SOPs as well as testing data sets to facilitate routine NGS implementation. Studies are continuing to evaluate the performance of short-read and long-read NGS platforms for virus detection. Cell-based standards are being developed to test the sensitivity of virus detection in cell substrates and cell therapy products. Database enhancement efforts are in place for increasing accuracy in AV detection and reducing follow-up of false positive signals.

At FDA-CBER, NGS data is currently being reviewed, in various applications: AV testing of cell banks, virus seeds and bulk harvests; genetic stability of viral vaccines; and cell substrate characterization. CBER's Advanced Technologies Team coordinates scientific discussions on new technologies, including NGS. CBER/OVRR highly recommends that sponsors request a technical working group discussion related to the use of NGS for vaccine safety and characterization. This is in the format of a non-regulatory meeting to discuss NGS implementation plans, aiming to reach a consensus before initiating lengthy and expensive studies. OVRR also provides consultation to the Office of Therapeutics regarding NGS applications for AV testing in gene and cell therapies. Furthermore, the CDER's Emerging Technologies Team is involved in discussions on NGS for biotherapeutics. Finally, the FDA's Center for Veterinary Medicine has initiated discussions with OVRR on NGS for AV detection for animal-based biologics.

There is general acceptance of NGS for AV detection. As a replacement for *in vivo* assays, NGS can provide defined sensitivity and breadth of virus detection and reduce animal use to meet the global 3R's objectives [23,24]. As a replacement for PCR assays, NGS can provide a single assay with similar sensitivity and broader virus detection. As an alternative method for supplementing or replacing the *in vitro* cell culture assays, NGS can be used for cell-substrate characterization, especially to address concerns for occult and novel viruses or to test virus seed and bulk harvest when the lack of effective neutralization of the vaccine virus could lead to assay interference. Additionally, NGS can be used as a readout assay to reduce assay time and for broader virus detection. However, it should be noted that NGS will generate frequent positive signals that will need follow-up to determine the biological significance of a signal for decision-making. NGS data may further help design a "custom" assay to determine if the signal is due to an infectious virus. NGS can be applied as a strategy to mitigate the risk of AV introduction and as a strategy to monitor/test the absence of AVs during production.

Regarding NGS in regulatory submissions, CBER generally expects the use of the WHO International Reference Panel or equivalent with cross-references or other details for the characterization of the material. Non-targeted NGS should be used for broad virus detection when replacing the general *in vivo* and *in vitro* virus detection assays and include details for sample preparation, such as sample pre-treatment, cDNA synthesis, and library preparation. SOPs and results should be submitted together with qualification and/or validation reports (the extent of data may be flexible based on the entirety of the submitted viral safety information) including virus spiking studies conducted in product-specific or relevant material. Submissions should also include the details of the NGS platform, sequencing, and results such as total reads, quality reads, number of reads analyzed, and method validation reports to demonstrate the throughput is sufficient to reach a satisfactory LOD for breadth and sensitivity of virus detection. Details of the bioinformatics approaches and pipelines used should be provided (as applicable), including host removal or the subtraction of the expected sequences along with accession or ID numbers for the relevant reference genomes used. Mapping data is useful for visualization. The virus reference database used should be specified with the version, together with details of the tools, program parameters and cutoff criteria for positive signals. Follow-up of positive hits should be detailed, including calling criteria for including or excluding hits and evaluation of positive results should be provided. The review of the various submissions is being used to develop a knowledge base and create a general format for expected information to be included in submissions.

Implementing NGS for AV detection is accepted and encouraged because it increases efficiency, reduces animal use, and provides superior specificity, LOD repeatability, and accuracy. Current cell substrate and viral safety guidelines and regulatory guidance documents (U.S. FDA [21], WHO [25], Ph. Eur. [12], ICH Q5A [R2] [1]) already provide the flexibility for using alternative approaches with broad virus detection capabilities and fit for purpose. There is still work needed for routine implementation. For example, current efforts are ongoing to optimize pre-treatment conditions to increase the sensitivity of virus detection in complex matrices. SOPs and reference data sets are being developed for broader NGS implementation, although other types of standards may still be needed. Database enhancement efforts are continuing to increase the accuracy of virus detection and reduce the follow-up of positive signals. CBER continues to support the in-house and external efforts toward NGS implementation.

### 2.4 World Health Organization (WHO)

**Ivana Knezevic** (WHO, Switzerland) presented on “WHO perspectives on the use of HTS for detection of adventitious agents in biologicals.”

Setting norms and standards and facilitating and supporting their implementation are core functions—entrenched in its constitution—and a unique strength of the World Health Organization (WHO). WHO norms and standards are composed of recommendations and guidelines (written standards) as well as measurement standards. This core work has enabled establishment and implementation of WHO international standards, facilitated by the expertise of the WHO Expert Committee on Biological Standardization (ECBS) and by an international network of eight WHO Collaborating Centers (CCs) and four custodian laboratories for Biological Standardization.

Established in 1947, the ECBS includes scientists from National Regulatory Authorities (NRAs), public health bodies, research institutes and academia. It provides decisions and recommendations based on scientific principles and public health considerations; and reports directly to the WHO Executive Board, which gives effect to the policies and decisions of the World Health Assembly. The outcomes of ECBS meetings as well as written and measurement standards are published in WHO Technical Report Series (TRS), well known as WHO blue books.

WHO CCs have a well-defined work plan and undergo re-designation every four years. One long-standing CC is the United Kingdom Medicines and Healthcare products Regulatory Agency (MHRA; which has taken on the duties in the area of biological standardization of the former National Institute for Biological Standards and Control [NIBSC]). MHRA develops and distributes over 95 % of all WHO international reference standards for biological products.

WHO is an advisory organization in nature that serves 194 member states. As such, it provides the principles for setting national requirements, leaving space for NRAs to formulate additional/more specific requirements, when needed. WHO guidelines are living documents that need to be further developed in line with the progress in scientific knowledge and experience. WHO also assists with the implementation of the guidelines into regulatory and manufacturing practice, although the responsibility for regulatory practice lies with NRAs, and the responsibility for manufacturing implementation lies with manufacturers. WHO facilitates these processes through global, regional, and national workshops involving regulators, manufacturers, academics, and other experts, and training and advisory groups. WHO considers guidance issued by other bodies, with the intention to complement them, without creating conflict.

Discussions regarding the detection of adventitious agents started in May 2007 within the WHO working group on cell substrates and cell banks. There was a need to move away from *in vivo* assays and introduce new technologies. It was agreed to encourage the development of new molecular methods, as published in the “Recommendations for Cell Substrates” [25] and the “Scientific principles for regulatory risk evaluation on finding an adventitious agent in a marketed vaccine” [26]. The First WHO International Reference Panel for AV detection in biological products using HTS technologies was established by WHO ECBS at its 79th meeting in March 2024 [10]. This Panel supports the wider use of such highly advanced and sensitive non-animal methods, with considerable benefits envisaged in accelerating testing timelines and thus expediting access to safe and affordable biological products. This reference panel with seven viruses supersedes the WHO international reference reagents with five viruses established by the ECBS in 2020.

WHO is now welcoming feedback on these reference viruses and preparing an implementation workshop to understand more about the practical aspects of these references. The panel is free of charge and provided for NGS qualification and validation studies for AV detection in biologics.

In August 2024, WHO issued a questionnaire for regulators aiming to understand the current situation regarding the use of HTS technologies in regulatory evaluation of biological products, identify areas where technical support is required for the use of HTS, and explore the expectations regarding the amendment to WHO recommendations on cell substrates. Responses were received from 16 regulators from different regions.

Twelve (75 %) NRAs have accepted HTS as an alternative to existing *in vitro* and *in vivo* AV detection methods for viral safety, test of biological products in marketing authorization, and/or clinical trials. Most countries are adopting or have already adopted HTS as an alternative for both *in vitro* and *in vivo* assays. The four countries not accepting HTS were India, Japan, South Korea, and South Africa. The reasons for not accepting HTS relate to a lack of a specific mandatory request or policy for HTS testing, validated methods, regulations, guidelines, and experience in the country.

Most countries that have already accepted HTS have authorized its use for replacing or supplementing the conventional viral safety tests in vaccines, biotherapeutics, and cell and gene therapy products. In China, the U.S., and Indonesia, this has only been authorized in vaccines. Countries have authorized this methodology for different steps of production manufacturing. The rationale and regulations/guidelines followed for the use of HTS varied and included international guidelines, national or regional regulations, and method validation reports submitted by the manufacturer.

Seven (47 %) participants/countries reported having considered the WHO recommendations provided in TRSs 978 and 993. Five countries responded that WHO documents need to be updated or elaborated and provided suggestions: detailed description of the state-of-the-art methods; information on high-quality reference viral genome database; a comparative table describing the pros/cons of each method for different manufacturing steps and types of raw materials; information about the reference panel; updated guidelines; a revision/update to align ICH and WHO documents. Among all participants, three (19 %) were unaware that the WHO International Reference Panel for AV detection by HTS was available at the time of the questionnaire but became aware of it during later discussions at this meeting.

WHO has organized several events in 2024 and 2025. These include expert consultations, working group meetings, implementation workshops on cell and gene therapy products and the evaluation of biosimilars, and the ECBS meetings in October 2024 and October 2025. A detailed report of the 80th ECBS meeting held on 7–11 October 2024 is published in TRS 1063 which is available, free of charge, on the WHO website [27].

### 2.5 Consortium on Adventitious Agent Contamination in Biomanufacturing (CAACB)

**Charles Swofford** (Massachusetts Institute of Technology [MIT], U.S.A.) presented on “Perspectives from the CAACB on the Current Benefits and Challenges of NGS Adoption for Viral Safety Testing”.

Viral contamination events have a very large impact on the patient due to potential safety reasons, but also due to product stock-out. They also have manufacturing, regulatory, and business consequences. Because of this, many companies have not publicly disclosed virus contamination events. Companies that have disclosed these events, rarely describe them in sufficient detail to be of significant value. To fill this information gap, in 2011, the Center for Biomedical Innovation at MIT set up the Consortium on Adventitious Agent Contamination in Biomanufacturing (CAACB). The goals of CAACB were to share experiences in the control and risk mitigation of adventitious agent contamination in biomanufacturing; to provide a forum for companies to network; to identify industry best practices; to provide opportunities to benchmark; to conduct collaborative research activities; to promote generation and application of new technologies (such as NGS); and to provide members early access to data and participation in analyses. These are achieved through interactive members-only workshops and other member-driven projects. CAACB members include CROs, Contract Development and Manufacturing Organizations, drug sponsors, and suppliers.

CAACB's flagship, living project, collects and analyzes viral contamination data in biomanufacturing. It is based on the premise that the confidential collection of industry-wide biocontamination data and subsequent risk analysis assessment would be a highly valuable “lessons learned” exercise for the industry and could guide companies in best practices to mitigate the risks that lead to these events. The project focuses on virus data from mammalian cell cultures, rDNA biopharmaceuticals, cell culture-produced vaccines, and other products at the manufacturing scale. CAACB members have provided Good Manufacturing Practice (GMP) and non-GMP manufacturing data since 1980. A 166-question survey was sent to each member, enquiring about contamination events, false positives, possible causes, and strategies for discovery, investigation, and decontamination. Data were anonymized, and results were published in 2020 [28].

The CAACB is aware of 29 viral contamination events reported directly to the consortium (n = 21) or publicly (n = 8). New insights from the survey were: (1) viral contamination events are rare based on volume (21 reported in 35 years) but not rare on a per-company basis (43 % of companies reported a viral contamination; 70 % reported a false positive event). These events were also expensive, with each investigation costing from one to ≥50 million dollars, excluding the cost of discarding materials, decontamination, and corrective actions. (2) The source of viral contamination was different in Chinese Hamster Ovary (CHO) cell cultures than in human and primate cells. CHO cell line contaminations were mostly with MVM and arose from media components (animal-derived serum). The contaminants in human and primate cells were mainly attributed to operators. (3) Virus safety testing has clear limitations, but when used in a targeted way, it can prevent virus spread in a facility.

*In vitro* virus tests gave a false negative result in five (out of 18) GMP contamination events, suggesting that these tests might not be sufficient to detect contaminations. PCR confirmed the virus in ten cases and identified it in 11 cases, while NGS was able to identify the virus in seven cases. Of note, these data have been collected since 1980, and NGS only started being implemented in the mid-2000s. Thus, over time, molecular-based assays have become critical for confirming and identifying contaminating viruses.

The contamination source was only directly identified in three (14 %) events. In all these cases, detection and identification of the source required molecular-based assay (biological amplification coupled to PCR), and there was a need to concentrate the raw materials. Molecular-based assays were further useful to help prevent the downstream spread. Out of the 21 total events, seven spread to the downstream process. Virus tests were not implemented as forward processing. In three cases a PCR test prevented downstream contamination. Furthermore, in most cases, PCR was a new technology, and it was developed to investigate the contamination. With NGS now considered as sensitive as PCR but more agnostic, it is likely that NGS could replace or supplement PCR. Based on the survey results, NGS started being used for investigations in 2005, and it now outweighs PCR as the most useful tool.

Another recently completed project at CAACB is the historical evaluation of the *in vivo* virus test and its potential for replacement with NGS [29]. CAACB members continue to report the use of the *in vivo* test: 94 % of them use it for cell line characterization, and ≈50 % use it for virus seed testing and lot release. Since 2000, CAACB members have performed more than 10,000 *in vivo* tests using more than 84,000 animals and more than 67,000 *in vitro* tests. This project aims to understand the value of the *in vivo* test.

In a previous study, *in vitro* tests were reported to perform better than *in vivo* [30]. However, there was no public record of historical *in vivo* testing data to understand if these results hold up in practice. CAACB collected historical data from 20 members on their experience with *in vivo* and *in vitro* tests and the use of NGS for viral safety.

Over the lifetime of using the *in vivo* test, all reports of a positive result on an *in vivo* test were also detected on another supporting assay. The false positive rate was around 0.2 % for the *in vivo* test and 0.02 % for *in vitro* tests. False positives in *in vivo* tests arose in eggs, mice, and in the MAP/RAP/HAP test. On average, these false positives took from one to three months to investigate and resolve, with one case taking over a year. The false negative rate was about 0.03 % for the *in vivo* test and 0.009 % for *in vitro* tests. Repeat assays were done for all 21 false positive cases, taking between 18 days and 3 months to complete. Furthermore, there are biological products for which *in vivo* or *in vitro* testing will not work (e.g., viral products with no neutralizing antibody). Thus, alternatives are needed for the *in vivo* AV test that are rapid, have improved sensitivities and specificities, and do not involve the use of animals for testing.

NGS is a potential alternative for viral detection. Short-read sequencing has been used to detect previously undetected viral contaminants in biological manufacturing and cell lines, as per examples provided in other talks. CAACB asked if member companies were interested in replacing or supplementing existing assays: 69 % of respondents are exploring replacing *in vivo* with NGS; 50 % are exploring supplementing *in vivo* with NGS; some companies have included NGS in Investigational New Drug filings. Regarding motivation to reduce animal use, >90 % of companies aim for greater breadth of detection and faster detection; 40 % aim for greater sensitivity, and 20 % reported being less expensive; one company reported having a company directive.

The technical challenges to NGS implementation are being addressed. AVDTWG studies are developing sample isolation and handling consensus, and Ph. Eur. is publishing a general chapter including validation guidelines. Bioinformatics requires in-house expertise, and there is a lack of guidance on setting the thresholds for these algorithms, but CROs have been filling this need. The lack of standards has been addressed by the availability of the WHO Reference Standards for AV detection. Nevertheless, NGS analysis time may be too long for some product modalities. Developing an NGS assay takes time. It is important to start now to save critical time, should an event occur.

### 2.6 European Federation of Pharmaceutical Industries and Associations (EFPIA)

**Noémie Deneyer** (GSK, Belgium, on behalf of European Federation of Pharmaceutical Industries and Associations [EFPIA]) presented on “EFPIA's perspectives on the validation and implementation of NGS for virus safety testing of biological products.”

EFPIA aimed to facilitate the implementation of advanced analytical NGS technologies related to clonality, cell line characterization, and viral safety for biotechnological products by sharing and leveraging industry expertise/experience, and best practices. The priority was to develop, promote, and provide guidance for NGS implementation as an alternative to current standards on viral safety testing under the scope of EU, U.S., ICH, and WHO regulatory landscapes. The EFPIA Supportive Group [31] included 16 companies. The main deliverable was a position paper on NGS for virus detection, published in June 2024, acting as a practical implementation guide and providing an industry position for discussion with regulatory authorities [28]. The different sections of the position paper were drafted between 2019 and 2024, and the process involved discussions with AVDTWG, CAACB, WHO, ICH, and EDQM. The position paper has been shared in workshops at PDA and CAACB.

The position paper is organized into four sections: an introduction to the characteristics of NGS technologies and comparison with conventional virus safety tests; a large section on validation strategies; a section on the analytical comparability of NGS with *in vivo*/*in vitro* safety tests; and a section on regulatory strategies for NGS implementation.

NGS for AV detection is considered a limit test. To control for impurities, there is a need to assess the specificity, the LOD, and the robustness during validation. For specificity, it is important to ensure that the test does not interfere with the matrix. For NGS, this means demonstrating the method's ability to detect a viral contaminant in a complex matrix and discriminate viral nucleic acids from the background. As positive controls, known spiked-in viruses can be used, with specificity being verified if the method identifies exclusively the viral sequences of the spiked virus or closely related viral species. A native sample can be used as a negative control (e.g., the matrix without the viral seed of interest), with specificity verified by the absence of signal from the native sample.

The LOD is the lowest amount of an analyte in a sample that can be detected but not necessarily quantified. It needs to be determined to demonstrate the method's performance in detecting low levels of contaminant viruses present in the sample. For NGS, LODs can be demonstrated using spiked-in samples at different concentrations. While NGS can detect a broad range of viral species, it is not possible to determine the LOD for all the detectable viral contaminants. The position paper proposes three ways to report LODs: worst-case LOD (underestimate of the LOD), list of virus-specific LODs (more informative), or range of LODs between best and worst sensitivity. Two types of model contaminants can be spiked: viral particles (using representative and fully characterized viral stocks) or infected cells (fully characterized) ([31]). Sample preparation can have a strong impact on method performance. Thus, spike model contaminants directly in the sample before extraction is recommended. Furthermore, sequencing depth can also impact the LOD. Method design should include validity criteria for the sequencing depth to be reached.

Robustness can be included as part of the validation or evaluated during the development phase. It should show the reliability of the analysis with respect to deliberate variations in method parameters. NGS-based methods are made up of different analytical steps. Therefore, risk assessment could be applied to identify critical steps and parameters. Then, the critical parameters identified should be deliberately varied to demonstrate the robustness of the analytical method. Examples of critical steps are sample extraction, library preparation, and sequencing. Robustness can be evaluated using artificially contaminated samples, such as the ones used to determine the LOD.

Regarding the comparability of NGS-based methods with existing virus safety tests, the ICH Q5A (R2) mentions that *in vitro* and *in vivo* methods can be replaced by NGS without the need for a head-to-head comparison. However, it is important to understand if validation is sufficient or if analytical comparability could/should be provided. The position paper provides a generic methodology to address comparability between two analytical methods in case of replacement. A pre-assessment should compare the intended purpose and targeted quality attributes, define the extent to which it is scientifically justified to compare the analytical results or decisions, and guide the design of a potential comparability study. During the pre-assessment, the following questions should be addressed.

• Is the intended purpose of the alternative method achieved by measuring the same type of product quality attribute as the reference method, or minimally is there a relationship between them?
• Do the two methods use the same signals or read-outs, or is there at least scientific evidence of a relationship or correlation between them?
• Is the test category of the alternative method the same as the reference method?

If an experimental comparability study is justified, it should compare the performance characteristics and analytical results of the two procedures and determine their ability to reach the same pass/fail decision for a given attribute. If the experimental comparability study is not scientifically justified or partially justifies the replacement, the risk assessment should be provided to the health authorities.

In the absence of an analytical comparability study, the replacement of *in vivo* tests can be justified if NGS is suitable for the intended purpose and if the method specificity, sensitivity, and breadth of detection have been demonstrated by validation studies. In the absence of reference data, LODs can be justified using comparative data already published in the literature. But these should consider that different NGS workflows can have different performances. The suitability needs should be justified based on the intended purpose and additional virus detection essay, including the overall control strategy.

For the replacement of the cell-based infectivity assays, EFPIA presents a more conservative position than the ICH Q5A (R2) due to the critical role of those assays in current viral control strategies [28,29]. Analytical comparability data can provide justification that analytical method performance is suitable for the intended purpose, but validation data alone may be sufficient to demonstrate extensive and sensitive virus detection in line with the needs outlined in the risk assessment. Analytical comparability may be limited because the used model viruses do not cover the entire scope of viruses. Model viruses used should cover worst-case scenarios (be detected at a low level), and stocks should be characterized in terms of genome copies and infectivity (different quality attributes). In the end, differences in terms of sensitivity and breadth of detection may be acceptable and need to be discussed in terms of the overall control strategy.

This project also involved sharing case studies between industries. Interactions with health authorities have been achieved via innovation-dedicated pathways or through ad-hoc, scientific, non-regulatory meetings with U.S. FDA-CBER or non-official interaction during conferences. Topics discussed included detailed NGS workflow, validation approach and results, nature and type of the spiking material, and available comparison results with *in vivo* or *in vitro* assays. Feedback was generally positive for the replacement of *in vivo* and *in vitro* by NGS. Questions raised by the authorities included details on data analysis, viral genome database used, validation of bioinformatical pipeline, and justification on the spiked model used and of the LODs. At least one application to replace *in vivo* assays was submitted and accepted.

A few needs from the industry remain to be further discussed: How to replace *in vitro* without analytical comparability? How to justify the LODs? What is the foreseen acceptance of the ICH Q5A (R2) statement regarding the replacement without head-to-head comparison in Europe, the U.S., and other countries? Are there perspectives on moving from a panel of orthogonal virus safety tests to a unique NGS-based assay?

### 2.7 Panel discussion: session 1

The session ended with a panel discussion moderated by Arifa Khan and Laurent Mallet and included all session speakers and additionally **Michael Wall** (Health Canada, Canada), **Juliati Dahlan** (National Agency of Drugs and Food Control, Indonesia), **Hadeer Abosalem** (Egyptian Drug Authority, Egypt), and **Hamida Begum** (National Drug Control Laboratory, the Directorate General of Drug Administration, Bangladesh).

The first discussion point concerned NGS validation of different cell lines and whether a validation report needed to be re-done for each cell line. Three scenarios were presented: cell lines of the same species, different species, or different strains of the same cell line. Changing the cell substrate for manufacturing should be considered a new product, but the question is whether the NGS testing would need re-validation if it has already been validated in another cell substrate. It was pointed out that verifying if the cell concentration is the same or similar between samples is an important first step because it can affect the background of host cell nucleic acids and LOD for viruses. This situation might happen more often when developing Advanced Therapy Medicinal Products (ATMPs), but this would probably need to be evaluated case-by-case, depending on how different the new cell lines or new clones are, how they were established, and the background content.

A similar comment was raised concerning changing the production cell substrate and NGS testing. The demands in early development differ from those for an approved product. Internal controls spiked in each sample would help evaluate. However, there are two different questions: whether the lack of interference would need to be demonstrated for another cell line of the same species when a cell control does not show interference, and whether a new cell bank would require new testing. It was noted that this again depends on what is in the cell line (e.g., a transgene) that could potentially interfere. The response would also depend on whether the approach is viromics, genomics, or transcriptomics and which internal controls can be spiked in. The question with the validation is whether it is possible to claim the same LOD with the new substrate.

A company viewpoint was stated: it is interesting to think about the validation of the cell substrate as a platform method. A full validation package is put in place for a given cell sample. If the method will be used for a new cell type, a risk assessment needs to be put in place, assessing the differences between cell lines, and doing matrix verification, to ensure that the LOD is reached. The trickiest question may be the background because it depends on the cell lines (i.e. number of cells). But this background can be assessed without redoing all the validation from scratch.

The second discussion point concerned the regulatory requirements for the verificiation of the murine, rat, and hamster antibody production assays (MAP, RAP, and HAP, respectively) since these are not always considered as *in vivo* adventitious virus testing methods. The general view is that NGS can be used to replace the MAP/RAP/HAP assays. The bioinformatics pipeline might differ if specific viruses can be targeted, which is also an advantage for sensitivity. There might be two ways of interpreting NGS data: a targeted bioinformatic approach and a non-targeted broad detection approach. However, the ICH guideline is generally open to using NGS to replace MAP/RAP/HAP assays. Checking specifically for these viruses in the bioinformatics platform is possible instead of checking against all viruses. This may allow a higher sensitivity, but determining the LOD and validating the assay would still be important. The NGS assay must be validated using the model viruses, even if the test is specifically targeting other viruses, because these may not be available to assess with NGS.

A third discussion point asked whether the virus reference panel would still be suitable when looking at cell therapy products, allogeneic cell therapy products, or others, since it is agnostic to the nature of the sample being tested. Model viruses have been selected based on different biological, chemical, and genomic properties. Thus, they are not species-specific, and they can be used in any background.

The fourth discussion point concerned the work on model viruses for a transcriptomics approach for cell bank testing since these model viruses would not apply in this case. It was indicated that the available reference viruses are being used to spike cells. In some cases, the viruses are added to the cell pellet, and then cells are lysed, as in a scenario where the cells express the virus. There are also ongoing efforts to develop cell-based standards. There are spiking studies using cell clones with a latent virus or with different levels of virus expression. These are being tested in a complex matrix. The current limitation is that each cell line only expresses one virus, and it will not be possible to develop cell lines for every of the model viruses, but it is also not feasible to have one cell line infected with different viruses. Cell-based standards will be provided to be used directly from the vial (no amplification or growth) because they have been characterized for expression. It was further added there is a fundamental difference between the validation of the transcriptomics approach and the viromics or genomics because the WHO reference viruses were selected based on different structures of the viral particles (capsid and envelopes) and genome configurations, which do not apply to the transcriptomics approach. It is possible to use synthetic RNAs or to derive cell lines transfected with synthetic RNAs expressing open reading frame (ORF) or ORF subsets for different viruses as a model without using naturally infected cell lines.

The fifth discussion point related to the WHO reference standards. These reference viruses are extremely good for validating from sample extraction through to identification, but they are too easy to detect from the bioinformatic point of view because they are very well-characterized viruses and have very good matches in the database. The potential contaminants may not be that close to reference sequences. Thus, there is a gap in bioinformatic validation, and there could be a false sense of assurance that the assay is working well. Standardized, synthetic sequences could be spiked into the bioinformatic datasets to challenge the bioinformatics pipeline. These could be developed based on evolutionary models to create sequences that are virus-like but not close to real viruses, and they would be an important addition to the existing standards. Having sequences to put into the matrix, independent of the extraction, would allow for testing at different stages of the process.

A group has tried heavily mutating sequences from the viruses in the WHO panel and adding them at the *in silico* stage. They have also removed the references from the database to evaluate if they could find distantly related viruses. A follow-up discussion point would be how to generate *in silico* reference data sets, which algorithms to use, and how to standardize them. These data could be used to test the bioinformatics step in addition to the other model viruses, but a standard would be useful. The challenge related to a bioinformatic standard reference is that the background of the raw data depends on the sequencing method. It is also important to consider the extent of diversity to be reflected by the reference data. The use of VirusPop, a database of simulated viral sequences [32], was suggested from the audience. It is based on evolutionary distance and provides a consistent list of fake viruses. It allows testing the capability of the pipeline to detect the distance sequences.

One of the challenges would still be if the sequences were so different that they represent an unrealistic challenge for the pipeline. Additionally, it is difficult to determine the actual sequence data and coverage. A suggestion would be simulating artificial reads from an already sequenced matrix. For example, this could allow evaluating if the pipeline correctly identifies a virus with a long homopolymer.

This specific feedback on how NGS works in practice is important, and users were encouraged to send feedback to anyone on the panel. This is also an important topic for further discussions in the working group and in other initiatives.

The discussion continued with perspectives from representatives of other NRAs in different countries who joined virtually.

**Juliati Dahlan** indicated that regulations in Indonesia have not yet required NGS. Experiences in evaluating the NGS have also been limited. However, the Indonesian NRA is following the ICH Q5A (R2) guideline, such that proposals using the NGS method for testing could be accepted. Applicants should provide evidence that the method has been validated, including demonstrating specificity, sensitivity, and any available comparability studies. So far, only one application has been submitted including NGS. This was an Oral Polio Vaccine 2 application. In this case, NGS was used for the characterization and monitoring of the genetic stability of the vaccine. This application used MAP/RAP/HAP for virus safety, but NGS would be recommended considering its limitations.

**Hadeer Abosalem** stated that the Egyptian Drug Authority (EDA) adheres to international guidelines. Following ICH Q5A (R2), EDA welcomes the use of NGS as an advanced method for the detection of adventitious agents. However, applicants should submit documents supporting the method's efficiency for detecting a broad range of viruses. EDA requires a comparative batch analysis, which does not have to be a head-to-head comparison. When using NGS as a replacement for conventional methods, applicants should submit the full method validation report, clear standard operating procedures (SOPs), and results to ensure the method is efficient and results interpretations are correct. So far, the EDA has not received any NGS data from any applicants. This may be due to a lack of applicant experience and limited validation development. Before implementation, the industry needs more support and training on the use of these advanced methods. Discussions with the manufacturers about replacing traditional assays have not occurred yet.

**Hamida Begum** indicated that in Bangladesh, NGS technology is very new, and it has not been introduced in Bangladesh yet. However, manufacturers are more advanced than the NRA, and some companies are trying to adopt the technology. In these cases, the NRA visits their facility and evaluates the documents, module, clinical trial data, and any testing results from foreign labs that are WHO pre-qualified. The NRA plans to implement this NGS technology in their lab, which currently has cell culture, microbiology, and *in vivo* testing labs. The NRA is trying to replace *in vivo* with *in vitro* testing, thus NGS would be welcomed.

The importance of manufacturers taking the lead to bring up a technology that will require building expertise among regulators was highlighted. Further encouragement is needed, and training or technical support to regulators would be useful. Discussions between manufacturers and regulators are useful even before submitting the data.

A cautionary note regarding batch-to-batch analysis or comparison was added. If the lab tests several batches side by side with both methods (*in vivo* or *in vitro* plus the NGS) for a while, it is likely that there will be no difference, and all results are negative because contamination events are very rare. Thus, both methods yielding negative results is not informative. It was suggested to use published data, model viruses, or materials from previous studies to evaluate the performance of the test in the desired matrix. Reference materials used to test the performance of *in vitro* tests can be reused to investigate NGS performance, allowing for a comparison between the methods. For the revision of the chapters in the Ph. Eur., a survey was also conducted among manufacturers and CROs in Europe. This showed that cases of contamination were never detected with the classical *in vivo* methods alone.

It was noted that important learnings could be obtained from the experience in the veterinary field, where financial constraints are higher and safety measures are more limited than in the human field. CAACB work so far has been contained within the CAACB membership. Thus, these studies would require that companies in the veterinary field to join the membership. This would be of interest to the consortium.

The regulators were asked to provide a brief overview of how they started introducing NGS as advice for those who are preparing to introduce the technology. It was indicated that regulators should not be reluctant and should start to gather all possible knowledge from the experiences shared in the conference and in publications.

**Michael Wall** noted that Canada is interested in reducing animal tests (although not bound to comply with the 3R's policies). When it comes to the replacement of animal tests, the most important point is the performance of the assay, and the new tests have to be at least as good as or superior to the existing ones. Canada has non-prescriptive regulations and works internationally instead of publishing Canadian-specific guidance documents. By law, they could accept almost anything that is scientifically justified, although there are limitations regarding harmonization across different regulators. An additional comment was provided that, although there is a directive regarding animal reduction in Europe, the main driver for introducing NGS is the scientific basis that traditional methods are insufficient. NGS is at least as good as PCR, and *in vivo* assays are not ideal.

## 3 Session 2: reference materials, NGS qualification and validation

This session was moderated by Sigrid De Keersmaecker (Sciensano, Belgium) and Johannes Blümel (PEI, Germany).

### 3.1 NGS transcriptomic assay

**Marc Eloit** (Pathoquest, France) presented on “Transcriptomic NGS assay of cells as a substitute for conventional virus testing techniques.”

When a virus replicates in cells, it accumulates messenger RNAs (mRNAs). These are the targets of transcriptomic analyses. mRNAs are common to DNA and RNA viruses. mRNAs are single-stranded, positive-strand molecules expressed during the replication of all viruses, including DNA viruses and most latent viruses. The level of expression is often very high, and sequences are short. Highly expressed cellular RNAs can be technically eliminated before sequencing, which increases the sensitivity of the detection of viral sequences. Thus, viral mRNAs are easier to detect than viral DNA. In addition, analysis of RNA profiles and/or metabolic labeling of nascent RNAs can differentiate replicating viruses from the carryover of viral sequences.

Pathoquest has previously shown that this NGS transcriptomic assay can replace *in vivo* tests [33] by comparing nine different viruses with corresponding cells in a head-to-head comparison with *in vivo* assays. The new study was set up in the context of the new ICH Q5A (R2), which allows NGS to replace virus-specific PCR assays and rodent antibody production tests without a head-to-head comparison. The study aimed to assess whether this NGS transcriptomic assay can replace targeted PCRs, MAP/RAP/HAP, and 9CFR assays for testing cells and determine the analytical sensitivity and the ability to detect distant strains in each targeted species. The study also discussed the performances for testing cell banks, bulk of recombinant proteins, and cell therapy medicinal products.

Since the tests to be replaced are targeted against a list of viruses, a list of target viruses was determined. A total of 78 different viruses were used for this evaluation. Different pools of test samples were made using synthetic RNA. The synthetic RNAs were produced to simulate sequences of distant strains. The cell pellet was lysed in a RNeasy extraction kit, spiking at 0.1 to 0.00001 RNA copies/cell. Two types of control samples were used: a negative control (MRC-5) and a positive control (a mix of two viruses, one with high expression and the other with low expression due to the dilution). Seven different RNA control mixes from the External RNA Controls Consortium (ERCC) were spiked in each test sample for a coverage ≥80 % for four of the seven targets compared to the spike in the positive control. Cell lysates were spiked with a different pool before RNA extraction. Then, samples were spiked with the ERCC library. NGS sequencing was done using NextSeq 500/550 High Output kit v2.5, single-read 150 nucleotides, at a depth of ≥65 million reads per sample. Virus testing used a targeted viral pipeline for the ERCC internal control and the targets, and an agnostic analysis for evaluation of the detection. The agnostic pipeline was published elsewhere [34], and it included filtering and de-hosting steps, assembly, and viral sequence identification by comparing translated contigs and singletons to the viral protein database (RVDB translated to protein). Positive hits were counter checked with the comprehensive protein database from NCBI. Each remaining hit was reviewed by an expert and reported.

For rodent viruses, NGS detected all close and distant strains at 0.1 copies per cell for all viruses tested, with high coverage. Regarding the human and simian panels, all closely-related virus strains were detected at 0.1 copies per cell, also with high coverage. For bovine and porcine viruses, all viruses were detected at 1 copy per cell, and all except one were detected at 0.1 copies per cell at high coverage. The LOD of this agnostic NGS test was thus estimated at 0.1 viral RNA copies per cell. The test detected 100 % of the 78 viruses, including human, simian, rodent, bovine, and porcine viruses. This LOD is also valid for distant strains aligning with the definition of these virus species from the International Committee on Taxonomy of Viruses, giving high assurance of reliability.

Based on these results, the study further discussed the predictive negative value of the result in case of negative results. Regarding the testing of cell banks, a risk analysis was performed based on published (non-comprehensive) viral RNA loads in infected cells. These sources reported viral load per infected cell ranging between 2 and 5 RNA copies per cell during active infection. In infectivity assays, the viral load is 10^4^ for herpes viruses during active infection but almost absent during latency (as an example). The worst-case LOD was 0.1 RNA copies/cell, equating to 1 infected in 1000 non-infected cells (10^−3^). This is coherent with experimental data using infected cells reporting an LOD between 10^−3^ to 10^−7^ infected per non-infected cell [33]. Thus, this level of analytical sensitivity obtained by an agnostic technique is sufficient to cover the risk for the characterization of master cell banks and late cell banks.

A discussion was further provided on testing the bulk of recombinant proteins produced in eukaryotic cells. Unlike bacterial cultures or chemical synthesis processes, eukaryotic cell cultures can support the replication and amplification of eukaryotic viruses. Regulatory guidelines require virological control specifically for eukaryotic cell culture-derived products. Bacterial or chemically synthesized products do not mandate viral testing of the bulk because the risk of viral carryover from raw materials is considered negligible. To detect over-amplifications that would have occurred during cell culture a viromic analysis on both intact or disrupted cells and supernatant could be done, as viral particles may be predominantly present in one or the other compartment. Sequencing must target both DNA and RNA. This is comprehensive, but this dual-target approach may dilute sensitivity compared to a transcriptomics approach focused solely on RNA. In addition, when the upstream of the process is inactivated (e.g., bovine serum), nucleic acid will yield a signal, and the genomics approach cannot differentiate it from replicating nucleic acids. An agnostic transcriptomics analysis on intact cells is thus preferred when intact cells are available (such as for biotech products, where cells are available at the end of the fermentation process).

Another application is for testing CAR-T cells for human herpes virus (HHV) 6 and 7 infection. A high proportion of adults are chronically infected with HHV‐6B, and many with HHV‐6A. The rate of detection of HHV-6 DNA in healthy adults is between 9 % and 90 %. Thus, testing by DNA PCR is not possible because it rejects a high number of batches of donors. This virus genome is episomal or integrated in a fraction of the body cells. In addition, the genome sometimes integrates in the germline, leading to the presence of this inherited chromosomally integrated HHV-6 in every cell of the offspring. This is the case in around 1 % of the human population. This latent virus can reactivate during the differentiation of CAR-T cells, leading to infectious virions [35]. *In vitro*, a population of HHV-6 “super-expressor” CAR-T cells (about 1 in 300-10,000 cells) possesses high viral transcriptional activity combined with an overexpression of the HHV-6 receptor (OX40). This background latency has been demonstrated by using peripheral blood mononuclear cells (PBMCs) from patients, lymphoid cell lines with latent herpes virus infection, and PBMCs from inherited chromosomally integrated HHV‐6 patients. This contrasts with active infection, where there is a wide range of transcripts, the most preeminent one often being the polymerase transcript (U38). The proposal regarding the strategy for testing is to use RNA-seq on cells that are cultivated, 2 weeks post-infusion time, typically 3–4 weeks after the beginning of the differentiation. This mimics the risk of reactivation after infusion *in vivo*. The acceptance criterion is “no detectable HHV6/7 RNAs after extended culture time”. The reliability (predictive negative value) of this test indeed depends on the LOD of the RNA-seq used. For 1 infected cell in 1000-10,000 non-infected cells, a satisfactory order of magnitude is 1 in 300-10,000, considering the ratio of cells that reactivate the virus.

In summary, the LOD obtained from non-regulated R&D testing of this GMP transcriptomics assay technology is estimated at 0.1 viral RNA copies per cell or, during active infections, at <1 infected cell diluted in 1000 non-infected cells. The assay detects all viruses, including distant strains across human, simian, rodent, bovine, and porcine species targeted by conventional tests (MAP/HAP/RAP, 9CFR, and extended PCR panels). Suggested applications include cell banks, cell therapy drug products, and bulk routine testing of recombinant proteins.

### 3.2 Matrix effects on LOD

**Bradley Hasson** (MilliporeSigma, U.S.A.) presented on “Matrix effects on LOD for NGS-based adventitious virus detection assays.”

The perspective of a CRO has several vantage points. CROs work with multiple clients from a cross-section of the industry testing different modalities. They address regulatory feedback for multiple applications of the technology, develop multiple validated methods for different purposes, and have NGS experts. CROs accumulate data on multiple products and backgrounds through spiking studies. Key elements for NGS biosafety and characterization testing include fully validated GMP methods, validated fit-for-purpose analysis algorithms, data integrity, secure data and storage and transfer, matrix suitability, and high-quality sample processing.

NGS is validated in a modular fashion for extraction, library preparation, sequencing, and bioinformatics. This approach confirms that each portion of the assay behaves as expected and offers flexibility for updating rapidly changing technologies. LODs for client-specific matrices are established through end-to-end analysis using known viruses to achieve ICH Q2 parameters. The algorithm conveys specificity and confirms it in validation, and LOD is established on a matrix-specific basis. The full validation package consists of generic validation plus any additional spiking studies in specific products.

Spiking studies use the representative reference viruses that are available through Biodefense and Emerging Infections Research Resources Repository (BEI Resources). Viruses are qualified by worldwide laboratories and standardized to be used in NGS adventitious agent testing applications. The model viruses contain different physiochemical properties aiming to represent different virus qualities and worst-case scenarios. In spiking studies, reference viruses are spiked at different concentrations into the test matrix which then undergoes the bioinformatic analysis. General guidelines followed at Merck are to use: modular validated methodology; client-specific matrix as representative material; a pre-study assessment of nuclease treatment; 3–5 representative viruses (WHO-CBER panel); 3–5 concentrations (depends on matrix and expectations); 2–3 replicates at each concentration. For the data analysis, a non-targeted approach is followed. It uses established criteria for detectability to mimic the analysis process (specificity, homology, fragment length, and number of reads required). Reproducibility is determined via replicates, and the required depth of coverage is defined. A significant increase in the number of spiking studies has been observed after the finalization of ICH Q5A (R2) at the end of 2023. In 2024 (to date) the number of spiking studies has doubled compared to the one in the previous year. These studies were performed to demonstrate LOD within the specific sample matrix. Typical samples seen were bulk harvests, master virus seeds, and master cell banks.

Sample preparation methods depend on the sample type and assessment type, with transcriptomics targeting RNA in the cell, genomics targeting total nucleic acids, and viromics targeting encapsulated nucleic acids. Merck uses Illumina-based technology for all NGS applications, which involves double-stranded DNA library preparation and downstream sequencing.

Matrix-specific LOD was determined using four representative cell lines for cell line validation and qualification. These were spiked with different concentrations of 3 RNA viruses from the CBER NGS Virus Reagents (RSV, FeLV, REO). Due to the breadth of detection of the NGS assay, the LOD of the assay was set based on the least sensitive virus detected. Data showed that the LOD of the NGS method in all cell lines tested was 10^4^ viral genome copies (VGC) per 10^6^ cells or 1 VGC/100 cells [36]. The LOD is verified in client-specific cell lines to confirm consistency with generic validation.

For non-cell-bank samples, the test matrix consists of enzymes, buffers, and solvents used in sample processing, a recombinant virus (in the case of a virus-based product), cells, and host cell nucleic acids (DNA and RNA).

It is important to consider that NGS analysis is non-targeted (agnostic). Generally, no exponential amplification is performed for the agnostic approach, so the background nucleic acid content matters greatly in terms of concentration. The WHO-endorsed NGS reference material (current CBER NGS Virus Reagents 5-virus panel) was qualified in adenovirus background only (10^9^ VGC/mL). Specific challenges were the presence and concentration of host nucleic acids, which can be removed using a nucleic acid degradation treatment (such as benzonase, DNase, etc.), and the presence, concentration, and genome length of viral nucleic acids, which have to be removed using specific viral nucleic acid depletion steps.

As a CRO, Merck has tested different viruses in different cellular backgrounds. The LOD may differ depending on the matrix. In a Vero background, the lowest LODs were obtained for COVID-19 (10^3^ VGC/mL). The highest LODs obtained were around 10^6^ copies (REO and PCV) in poxvirus of high concentration. In human background, for adenoviruses of different concentrations, LODs were generally higher (10^5^-10^6^ copies). In an avian background, which was clean in terms of residual nucleic acid, the highest LODs were 10^4^ copies for REO and PCV and with all others at 10^3^ copies. In insect background (containing baculovirus at a high concentration), LODs were up to 10^6^ copies even after benzonase treatment. The same generic NGS method helped qualify the reference material. These results suggest that one method might not be suitable for every matrix.

A case study was presented to assess benzonase endonuclease's effects in non-viral material. In the U.S., there was an issue with contamination of the fetal bovine serum with influenza, H5N1. Merck has screened new lots of fetal bovine serum using NGS for influenza. Viruses were spiked at high concentrations (10^6^) and tested for the effect of benzonase treatment. Results showed that benzonase treatment reduced the background and obtained better LODs compared to non-treated samples.

In summary, cell lines behave relatively consistently in terms of LOD using representative viruses. Complex matrices (e.g., bulk harvests, master virus seeds) may show differences in LOD depending on matrix composition and may require matrix-specific processing steps. A key variable seems to be recombinant virus type and concentration. “Prior Knowledge” may help predict LOD in certain matrices, but one representative complex matrix does not currently exist. Spiking studies using matrix-specific representative material will continue to be performed, although this can likely be streamlined through LOD verification procedures instead of full spiking studies. Key questions remain on the minimum acceptable LOD and what a true infection would look like at the point of testing, which is likely best addressed on a product-by-product basis.

### 3.3 AVDTWG spiking study 4 – targeted analysis

**Valeria Zanda** (Merck, Italy) presented on “Adventitious Virus Detection Technologies Working Group spiking study 4 - Evaluation of long-read sequencing for adventitious virus detection in a low complexity viral background.”

The mission of the AVDTWG is to facilitate the use of advanced technologies (such as NGS) for the detection of AV in biologics by providing an informal scientific forum for knowledge exchange, scientific discussions, and collaborations among scientists across different organizations. To achieve this goal, subgroups have been established that address different topics related to the laboratory and bioinformatic steps in the NGS workflow. In parallel, collaborative studies are organized based on short-read or long-read technologies. Spiking study 4 aimed to evaluate the application of long-read sequencing for AV detection. The model used was a high-titer adenovirus 5 (Ad5) spiked with the five CBER NGS Virus Reagents. The study involved 12 organizations, and it is expected to be completed by the end of 2025.

The long-read sequencing technology used was Oxford Nanopore sequencing [37]. At the time the study started, there was limited experience with this type of technology, and it was decided to evaluate this new technology for sensitivity and specificity for virus detection. The high error rate was a known constraint of this platform at the time. Thus, a simple matrix was used for the study. Other more complex backgrounds were planned to be analyzed as part of spiking studies 5 and 6.

The design of spiking study 4 was similar to spiking study 2B for comparing the results with the short-read technology [38]. The starting material was composed of a high-titer Ad5 (10^9^ genome copies in 200 μL). This background was spiked with the 5 model viruses. All participants tested two spike levels (10^4^ and 10^6^ genome copies). They could perform the study using a common or individual protocol and conduct targeted and non-targeted bioinformatics analysis.

A common protocol was developed and shared within the group. This included a common method for sample preparation including viral stock dilutions and preparation of the spiked samples. The extraction phase recovered both RNA and DNA, and a reverse transcription step was applied to obtain double-stranded cDNA. The participants defined library preparation, sequencing, and data analysis individually. The following points were considered: nucleic acid content is low, and the workflow aimed to preserve long viral fragments to optimize for high viral recovery. Since the technology is evolving fast, different versions/updates of the chemistry and the software needed pipeline adaptations.

Results from the targeted analysis were presented for the Ad5 background and the two spike levels. The total number of reads obtained ranged between 8000 and 14 million reads. The mean read quality ranged between Q7 and Q17. All labs were able to detect all viruses at 10^6^ genome copies, and most labs detected all the viruses at 10^5^ genome copies. It was noted that the long-read technology can detect circular viruses. All viruses except PCV1 were observed at 10^4^ VGC by most labs.

A few lessons learned during this study were shared with the audience. This technology is rapidly evolving with frequent updates of chemistry and software, which needed to be adjusted in this study for comparing results. In addition, variability in the flow cell from batch to batch was observed. The workflow was set up to evaluate the impact of the starting material. The protocol needed to be adapted to preserve long nucleic acids. The impact of the quality criteria for base calling on sensitivity should be further investigated. There were full-length reads longer than the viral genome size, which might be chemistry artifacts. Finally, the bioinformatics pipeline needs to be adapted to work on long reads.

### 3.4 Head-to-head comparison of NGS and conventional assays

**Alison Armstrong** (Merck KGaA, Germany), on behalf of the National Institute for Innovation in Manufacturing Biopharmaceuticals [NIIMBL] PC3.1-305 project team) presented on “Head-to-head comparison of NGS with *in vivo* animal assays and *in vitro* cell culture assays for adventitious virus detection”.

NGS has demonstrated capabilities for the detection of known and unknown viruses in biologics, without needing prior sequence knowledge. This project aimed to obtain NGS data for decision-making regarding the suitability of NGS applications for AV detection in biologics. Points addressed included replacing animal-based viral adventitious agent testing, developing tools to support novel *in vitro* adventitious agent testing, and identifying new *in vitro* release tests to replace animal-based release tests in vaccine manufacturing quality control. The key project aims were to: (1) evaluate NGS virus detection in a complex biological material and directly compare with the *in vivo* animal assays and the *in vitro* cell culture assays using the same spiked samples for testing in all of the different assays; and (2) enhance NGS bioinformatics for known and novel AV detection by refining the Reference Virus Database (RVDB) for increasing specificity and accuracy of virus detection and building automated pipelines for enhancing efficiency of NGS bioinformatics work. The project involved collaborators from the U.S. FDA/CBER, Merck/MilliporeSigma, and GSK for the NGS and associated bioinformatics analysis work, and from the U.S. FDA/CBER and the University of Delaware for the database and bioinformatics enhancements.

It is generally presumed that NGS is superior to the *in vivo* and *in vitro* assays regarding the breadth of virus detection, and NGS can have a similar sensitivity of virus detection to PCR assays. This study was specifically designed to challenge the NGS workflow and to maximize detection by the biological assays. This was achieved by selecting viruses known to be infectious in at least one of the target cell lines in the *in vitro* assays or one animal species in the *in vivo* assays. To achieve this goal, only two of the 5 CBER NGS Virus Reagents [39] were used for spiking to evaluate AV detection, and therefore the breadth of NGS could not be evaluated compared to the other assays in this study. The two RNA viruses used in the study were human respiratory syncytial virus (RSV; single-stranded, enveloped virus) and mammalian orthoreoirus type1 (REO; double-stranded, non-enveloped virus). However, based on the literature and the AVDTWG 2B study [38], it is known that NGS could detect viruses that would not be expected to be detected in the *in vivo* and *in vitro* assays (e.g., PCV-1, EBV, FeLV).

Selected human RSV and REO were already characterized for virus copies/mL, infectious titer, host cell DNA, and AV. In-house digital droplet PCR (ddPCR) assays were available in the CBER lab for these viruses. The sample type selected as the matrix for the spiking study was a complex CHO cell-based bioreactor material. Prior to the study initiation, this matrix was evaluated for sterility and tested for mycoplasma, host cell proteins were determined by BCA (Bicinchoninic Acid) protein assay and ELISA (Enzyme-Linked Immunosorbent Assay), and host cell DNA was quantified via ddPCR assay. It was decided that no toxicity study was needed due to previous experience. Twelve tenfold serial dilutions of each virus were made and spiked into a fixed volume of matrix. Samples were blinded and stored at −80C prior to distribution. Aliquots were made for one-time use per experimental setup per assay without a freeze-thaw. Six spiked samples and one unspiked control were selected for testing in all assays by all study participants. The six samples were selected based on a pre-study to determine the range of infectious titer and genome copy number, which would produce a positive result in the test assays with at least one virus. The same volume was tested by ddPCR, NGS and *in vitro* assays for a head-to-head comparison. The same material was tested *in vivo,* using a tiered strategy to minimize the number of animals, starting with the lowest dilution. Volumes for inoculation of each species were based on the protocols for the compendial assay.

A 28-day *in vitro* cell culture assay was performed using Vero, MRC-5, and CHO-K1 cell lines. The two participating labs used the same experimental setup per the compendial assay. Observations for cytopathic effect (CPE), hemagglutination (HA), and hemadsorption (HAD) were noted during the study period, as applicable. *In vivo* assays were performed in suckling mice, adult mice, and embryonated hens' eggs. The age and number of mice and eggs, volumes and routes of inoculation, observation times, and readout for clinical signs or death were done based on the compendial assay. A subpassage was included for suckling mice and embryonated hens’ eggs. NGS was done using short-read technologies. Each lab used its own protocol for the entire workflow, including the bioinformatics pipelines. To compare results for virus detection, all used the same accession numbers of the reference virus sequence for the targeted analysis and RVDB for the non-targeted analysis. For the ddPCR, results from the pre-study were used to determine the sensitivity of virus detection.

The overall results show that, with NGS, all (3) labs detected both RSV and REO in the complex background of the unprocessed CHO cell matrix, although a difference in sensitivity of virus detection was seen. A review of the protocols indicated this was due to different NGS workflows (pre-treatment to reduce host cell nucleic acids in the sample may be important for enhancing virus detection). NGS showed a similar sensitivity of virus detection to the ddPCR results obtained from the pre-study. Results of the *in vitro* cell culture assays (2 labs) showed similar results with REO, using an independent source of cell lines, reagents, and observation of results for the readout assays (CPE, HA, HAD). Differences in virus detection were seen with RSV. As expected, a higher sensitivity of virus detection was seen in the cell culture assays compared with NGS. With *in vivo* assays (1 lab), a positive result was seen with REO based on subpassage. However, this was due to a modification in the subpassage for suckling mice. This would require high levels of specific investigatory work in standard practice, but this was limited due to the ethical constraints of using mice.

Three principal complementary approaches are used to control for potential viral contamination of biotechnology products, ICH Q5A(R2). The key method to control for contamination is the *in vitro* infectivity test using selected cell lines followed by end-point analysis (CPE, HAD, and HA). This study provides experimental data and confirms that levels of variability of detection between laboratories are likely based on cell line choice, pre-treatment of test materials, and end-point analysis during the test period. RSV and REO were detected in replicate samples by CPE, HA, HAD, or any combination of the endpoints in any one of the three indicator cell lines. All positive and negative controls performed as expected. Both viruses were detected by CPE, and only REO was detected by HA using rhesus monkey cells at two temperatures. The study shows the variability in the results and highlights the limitations of virus detection using the traditional *in vitro* methods, which further provides evidence that supplementary or replacement of this assay using NGS should be considered.

This is the first study to directly compare NGS with both the *in vivo* and the *in vitro* virus detection assays, using the same sample material in all the tests. The results support the recommendations for using NGS in the updated ICH Q5A(R2) guideline on Viral Safety Evaluation of Biotechnology Products Derived from Cell Lines of Human or Animal Origin [1]. NGS can be used as a replacement for *in vivo* and PCR assays. It can provide defined sensitivity and breadth of virus detection, and it aligns with the 3R's initiative to reduce animals for testing. NGS can have similar sensitivity to PCR assays and has a broader virus detection in a single assay. It can also be used as supplementary or replacement for *in vitro* assays, particularly in cases with concerns for occult and novel viruses such as in cell-substrate characterization, in case of assay interference due to lack of effective neutralization of vaccine virus, or as a readout to reduce assay time. This study provides head-to-head data to support the suitable use of NGS in biologics and was submitted for publication during the preparation of this report [40].

Additional work is being developed for routine implementation. The group is optimizing NGS for broad virus detection in complex matrices by spiking studies using the seven viruses in the First WHO International Reference Panel and 2 well-characterized virus-infected reference cell lines developed by the CBER lab. NGS bioinformatics is also being optimized to enhance the efficiency of database generation and accuracy of data analysis for adventitious and endogenous virus detection. Ultimately, the aims are to develop protocols, strategies, and NGS datasets to establish the technology broadly and for routine use.

### 3.5 RVDB

**Pei-Ju Chin** (U.S. FDA, U.S.A.) presented on “Refinement efforts on CBER's Reference Virus Database (RVDB) to enhance accuracy and specificity of virus detection.”

The need for RVDB arose from the limitations of public databases: 1) NCBI Viral Genomes Resource (RefSeq and Neighbors) includes complete, annotated viral genomes, but other viruses with only partial genomes and endogenous retrovirus sequences are under-represented. It is limited in sequence diversity with one record per virus species for RefSeq (some exceptions) and some diversity in Neighbors, but not all-inclusive; 2) NCBI NR/NT (non-redundant nucleotide collection) includes additional viral diversity with full viral genomes and partial viral sequences, but it also includes an abundance of cellular sequences and uncharacterized sequences. The large number of non-viral sequences can confound the detection of viral signals. Many sequences are mis-annotated (cellular sequence annotated as viral and vice versa); 3) NCBI Protein database (non-redundant protein sequences) only contains complete protein sequences, so partial virus sequences that cannot encode proteins are not represented.

In-house laboratory efforts toward developing a comprehensive RVDB was initiated in mid-2013, in consultation with the AVDTWG and NCBI. RVDB development employed semantic data mining of selected GenBank divisions, NCBI Viral RefSeq/Neighbors, and NCBI Third-Party Annotation to include all viral, viral-related, and viral-like sequences, including endogenous viruses and retroelements, with the additional goal of having a reduced non-viral/cellular content to improve virus detection specificity by NGS. The initial RVDB production workflow was published in 2018 [11]. A semantic-refine filter is applied, which includes keywords, (currently ≈700), selected based on knowledge acquired from manual curation.

RVDB version 29.0 was released in July 2024 and includes over 10 million sequences in the unclustered version and 1.1 million sequences in the clustered version [41]. It is provided in four different formats to adapt to various applications.

• U-RVDB fasta file: Unclustered files containing all viral sequences with redundancy. High computation power is demanded. Suitable for virus detection by BLASTn/nhmmer.
• C-RVDB fasta file: Clustered files, where sequences that share 98 % similarity are collapsed to one representative sequence per each clade. Low computation power is demanded. Suitable for virus detection by tblastx.
• SQLite DB Script: Creates the entries (fasta header and the corresponding information) for advanced bioinformatic pipelines/workflows.
• Proteic RVDB (provided by Institut Pasteur [42]): Hidden Markov Model profile of viral protein domains. Suitable to search for unknown viruses with remote homology by hmmsearch/hmmscan.

The collection of sequences in RVDB is subset directly from NCBI GenBank, viral RefSeq, and Third-Party Annotation without any modifications. Therefore, potential issues are inherited, including poor quality of sequence (e.g. poly-Ns in SARS-CoV-2 sequences), sequencing vector carryover (vector/adapter/linker/primer … etc), mis-annotation of non-viral sequences as viral, and vice versa, or flanking host sequences associated with endogenous retroviruses. Pipelines and strategies for overcoming these issues have been developed in the past years. The keywords list is updated based on discussions within AVDTWG subgroup C. Sequences are discarded if they correspond to irrelevant viral sequences (sequences indicating untranslated regions (UTRs), satellite sequences, phages, viroids, sequences with no virus defined in the environmental sample division) or if they are mis-annotated viral sequences (stealth viruses, non-viral sequences verified by manual curation, “TY” and “Pol” terms used to name clone, host/cellular “IAP”).

As part of the strategy to improve virus detection accuracy, an automatic annotation pipeline to indicate non-viral regions in RVDB has been developed in collaboration with Trent Bosma (CBER) and Madolyn MacDonald (University of Delaware). RVDB is used for NCBI BLASTn against their in-house, knowledge-based, non-viral dataset (rRNAs, mitochondria, protozoa, bacteria, and phages). Any homologies retrieved are annotated in RVDB with the accession number and coordinates of the non-viral region to allow for unexpected hit follow-ups (resulting from the cross-reactivity of non-viral segments) and database masking to reduce false positive hits.

The RVDB has also been refined behind the scenes to transition to Python 3 scripts and consolidate the number of scripts. A taxonomy-based phage cleanup pipeline was implemented to supplement keyword-based search. A filtration pipeline for SARS-CoV-2 sequences was developed to detect and filter out Poly-N sequences. Due to the increasing size of the database and the burden from flooding with large and redundant SARS-CoV-2 sequences, a new strategy for collapsing and generating C-RVBD has been developed. In this strategy, SARS-CoV-2 sequences with ≥1 Poly-Ns are discarded. Sequences with <1 % Poly-Ns are then aligned to the ancestral Wuhan strain (NC_045512.2). Sequences with ≥98 % identity are then represented by the Wuhan strain; those with <98 % identity are collapsed via the general MMSeqS2 algorithm used for the C-RVDB. RVDB without SARS-CoV-2 redundancy decreases the computational burden for NGS bioinformatic analysis by reducing the number of sequences by 5220-fold and the database size by 51,290-fold. Although this sacrifices some diversity, it still keeps the variants of concern. The refinements to RVDB were published during the preparation of this report [43].

The RVDB user community has been growing. In 2023, there were more than 5000 users worldwide. Efforts to update and refine RVDB are continuing. Quarterly updates are issued based on NCBI's release schedule. Ongoing and future work includes the annotation of non-viral and viral but with host content and endogenous (retro)viruses and the improvement of the user experience for the RVDB website based on the feedback retrieved via the routine user surveys. Work is also ongoing to optimize RVDB for modern CPU architecture (multi-processing cores) to accelerate the production pipeline. Furthermore, the team is working on incorporating artificial intelligence (AI)/Machine Learning (ML) to assist the manual RVDB review process. Although BERT (a Large Language Model) has been implemented since v26.0, models with larger parameters (e.g., Google Gemini, Meta LLaMA, and OpenAI GPT4) will be evaluated for their potential for higher accuracy.

### 3.6 NGS as an alternative to *in vivo* testing for an influenza vaccine

**Alice Alston** (AstraZeneca [AZ], UK) presented on “Validation, regulatory approval and implementation of NGS as an alternative [to] *in vivo* testing for live attenuated influenza vaccine.”

The use of NGS can be appropriate to identify adventitious agents in different circumstances: for infectious products or intermediates where a neutralizing antibody is unavailable for culture-based methods; for cell line screening to replace animal studies and *in vivo* testing; as a broad virus screening assay to complement or round out the safety profile of the test agent; for product release testing as an alternative method to confirm safety profile; in instances where traditional assays would not be expected to identify a potential contaminant of interest or no traditional assay exists; as a replacement for *in vivo* and *in vitro* methods (with appropriate qualification/validation testing), because it is faster and consistent with 3R's principles to reduce animal use.

Before starting, AZ evaluated the considerations for the use of NGS. Virus identification through NGS requires follow-up investigation since NGS detects virus nucleic acids but not infectious particles. The information gained through sequencing should be able to guide the investigation path to look at virus identification, whole or partial genome, and known replicative intermediates. The method quality was also a consideration. It was important to establish method suitability and validity criteria, including sample preparation, assay limits, and process controls. Also needed to consider how the data was processed and stored, what would be the bioinformatics approach and data interpretation in the context of the sample tested.

Because this work was developed with Merck, the NGS assay was validated in a modular fashion, as described by Bradley Hasson (Section 3.2): extraction, library prep, sequencing, and bioinformatics. LODs were determined through product-specific qualifications. The five viruses in the WHO reference reagents were used. The goal was to replace the *in vivo* animal model testing for AV. The test sample was bulk harvest material for quadrivalent influenza vaccine, with a matrix of allantoic fluid split into four separate strains and lots: H1N, H3N2, B Yamagata, and B Victoria. The vaccine is manufactured using Specific Pathogen Free eggs as per Ph. Eur. 5.2.2 [44] and U.S. Department of Agriculture memorandum 800.65 [45].

QC lot release testing is performed at all stages of the manufacturing process. The testing is designed to assess the safety, purity, identity, and efficacy of the drug substance/product. Testing follows Ph. Eur. 2.6.16 [16] and U.S. Guidance for Industry [21]. *In vivo* testing, as specified in Ph. Eur. 2.6.16 [16], is completed on every lot manufactured. This assay was subject to high invalid rates due to inherent variability within a biological model, egg hardiness, etc. NGS was chosen as a suitable alternative that can deliver and scale with quality, speed, and efficiency, supply to market with a repeatable, robust assay, accelerate digital and technical innovation, and remove animal testing in line with AZ's 3R's program.

Method suitability was evaluated using one replicate for 10^6^ genome copies and two replicates for other spike levels (10^4^, 10^3^, 10^2^) per sample type. Hits for each spike level were tabulated and assessed in the context of a total number of hits and hits per million. The number of hits was high for 10^6^ spiked samples and decreased as the spike level decreased, down to little to no hits seen in the 10^2^ spiked samples. Detectability was set as positive if detecting at least one hit. All assays and controls passed the validity criteria as expected, and no hits were detected in the unspiked samples. Detection was positive for four viruses at 10^3^ genome copies/mL and for all viruses at 10^4^ genome copies/mL.

The spiking study allowed to conclude that NGS was able to detect representative virus spikes at suitable limits of detection, in line with previous matrices published through worldwide studies. Results were within the expectations regarding LODs between 10^3^ and 10^4^. The higher limits of detection in REO and PCV were not unexpected given the nature of the viruses (REO: double-stranded RNA virus, PCV: very small single-stranded DNA virus). Read numbers ≥100 million reads per sample were ideal for hit and LOD based on the “hits per million” assessment. The minimum read count was set at 130 million for this matrix.

AZ did not have any technical discussions with the FDA, EMA or any other agency prior to regulatory submission, which would have been valuable in hindsight. The regulatory submission approach included a summary validation package provided by Merck. A final summary report was compiled by Merck and AZ detailing the overall product-specific qualification approach and all the data. AZ had set up a contamination control assessment of manufacturing and QC labs to show how adventitious agents could not be introduced during manufacturing or testing. The full risk assessment for the introduction of NGS as an alternative to *in vivo* included: the contamination control of adventitious agents; a comparison of NGS versus *in vivo*; risks of switching to NGS; an assessment of the product safety profile and discussion of other safety tests completed; an investigation approach for a “positive hit” in routine testing. The overall summary was that NGS offered a more robust and sensitive method, along with other registered safety assays, for the detection of adventitious agents.

Examples of questions received from the health authorities were presented. These included requests for detailed description and justification of the algorithms and criteria for the definition of a hit/positive viral hit; questions related to the matrix and materials used for the spiking studies; a comment on the LOD for REO and PCV being higher than the others, and a recommendation to continue improving the LOD for those families; questions on the rationale to determine the specific viral signals as not being considered significant; questions relating to the detection of influenza strains different to the expected live attenuated influenza vaccine (LAIV) strains during the BLAST results of the pooled harvest fluid. These influenza sequences were ruled out of the analyses due to the following reasons: for B strains, the 6 internal genes of the backbone of the vaccine (B/Ann Arbor/1/66) are combined with 2 external genes of the circulating virus to produce LAIV, so other B strains could be detected; for A strains, the two different strains identified during BLAST analysis had regions >100 bases with high levels of homology to the strains being analyzed, thus the detection of other sequences was expected given the conserved approach used for identification of potential virus sequences. Finally, health authorities also added a request to submit plans for updating the validation studies for NGS virus detection as part of continuous improvement and life cycle management.

Questions received were from the EMA, Swissmedic, and the U.S. FDA. All questions were responded to within the boundaries of active submissions under review. So far, markets that have approved are EMA, MHRA, Swissmedic, the Ministry of Health in Israel, Health Canada, and the FDA. NGS is currently under review in Mexico, Taiwan, South Korea, and Australia, and discussions are ongoing with Japan to understand if NGS will be accepted or if the *in vivo* assay is still the chosen method for adventitious agent detection.

NGS is now the preferred option for viral adventitious agent detection in LAIV, although *in vivo* is still used for specific lots for markets where NGS is not yet approved. NGS has been used routinely as part of the lot release program since April 2023. Since then, there were 0 % invalid assays (in *in vivo* this could be as high as 50 %) and zero positive hits for investigation. The turn-around time (TAT) for NGS is 32 days, compared to 60 days for *in vivo*, corresponding to a 28-day TAT saving. NGS is more costly than *in vivo*, however, this is balanced by a reduced TAT, fewer invalid assays, and fewer investigations for unconfirmed “Out of Specifications”.

### 3.7 Panel discussion: session 2

The session concluded with a panel discussion moderated by **Sigrid De Keersmaecker** and **Johannes Blümel** and involving all session speakers. The first question was addressed to **Bradley Hasson** about the difficulties around the insect cell harvest, even after benzonase treatment. He explained that, although the benzonase treatment works, the matrix contained high levels of baculovirus that cannot be removed. This ultimately resulted in a higher LOD.

The second question, to **Valeria Zanda**, was whether the variability in sequencing depth between labs (from 8000 to 40 million) could be correlated with the input library. She replied that there are different factors for consideration. One could indeed be the input library, but it could also be related to variability among different batch-to-batch flow cells for the sequencing or the quality/number of available pores in the cells. Another point to consider is that different labs may use different chemistries, base calling algorithms, or software versions. Once the complete data package for the study is available, it may be possible to identify critical points for consideration when applying this technology. A follow-up comment suggested that ML models may be used to identify the features impacting the output quality.

The third question was directed at **Alice Alston** and **Bradley Hasson**, regarding the higher LOD obtained for REO and PCV. Since PCV has the smallest genome of the whole panel, could this higher LOD be related to the matrix? Bradley Hasson confirmed that they tend to see higher LOD with these two viruses compared to other viruses. A follow-up comment suggested trying to open the double-stranded RNA to improve sensitivity for REO.

The panel was asked about the comparison of the sensitivity of virus detection between PCR and NGS. In the data shown, there was variability due to the different matrices. It would be interesting to verify if these changes in sensitivity correlate with the PCR. If PCR and NGS are comparable to each other one would expect that a difficult matrix is verified in PCR as well as in NGS. **Bradley Hasson** answered that since PCR involves exponential amplification, it generally allows amplification of the target above the background, whereas, in NGS, the background is higher, worsening the LOD. **Arifa Khan** added that, in the study presented by **Alison Armstrong**, a ddPCR assay was done to identify the range of samples for testing by NGS. In this case, the sensitivity was close between ddPCR and NGS. It is also important to be aware that the sensitivity of the PCR assays also depends on the background, which can inhibit the reaction. These experiments are challenging NGS testing for determining the sensitivity/LOD.

The discussion followed with a question regarding whether it is possible in transcriptomics to attribute the sequence to the species level. **Marc Eloit** replied that this depends on the sequence. The pipeline they used returns the lowest common ancestor. If the sequence is shared between different species, it will return the genus. If it is shared between different strains, it will return the species, etc.

A question for **Pei-Ju Chin** concerned the example of HIV being confounded with mitochondria. Would it be possible to mask these file sections to avoid retrieving hits from them? **Pei-Ju Chin** clarified that the annotation sheet includes the accession numbers and coordinates of any identified non-viral regions. The annotation does not imply a certainty that this region is non-viral, just that it has a high similarity to a non-viral sequence. Thus, the user must decide how to use this annotation, depending on the risk management strategy. **Arifa Khan** added that there is confidence that sequences annotated as non-viral are, in fact, non-viral. The bioinformatic analyses and BLAST comparisons performed by Trent Bosma use non-viral datasets that do not suggest any homology to viral sequences. However, RVDB does not take the responsibility of masking those sequences because that would require demonstrating that no viral sequences have also been masked.

**Laurent Mallet** commented that it is always challenging to make a head-to-head comparison. There will always be viruses that are better detected by cell culture or *in vivo* than by NGS. However, the comparison exercise should also consider the breadth of detection of NGS. This requires a balanced interpretation of the results. He further asked about REO virus results. **Alison Armstrong** confirmed that results were positive for REO by CPE and HA and not HAD, but only using rhesus monkey red blood cells. The strain used was the human type 1 REO Lang strain; thus, it is cell culture adapted.

A question to **Bradley Hasson** enquired how the cell type background was assessed in the platform method validation for the cell-based sample. A cell line can come with other backgrounds (for example Sf9 can come with rhabdovirus). **Bradley Hasson** answered that, when running NGS, different sequences are obtained, and it is possible to place them in the context of what is being analyzed. Thus, rhabdovirus sequences were observed in Sf9-spiked samples, as supported by the literature, but they were not detected in unspiked samples. He added that they do not do host cell filtering, but they call out sequences that, based on the literature, are expected to be found in the context of that cell line.

Another question followed on whether the thousand-fold LOD difference in matrix effects is entirely due to virus production. **Bradley Hasson** replied that this depends: sometimes there are host cell sequences, but these can be removed with treatments such as benzonase or filtration. The data shown here were already optimized for host cell sequence removal, so these sequences were coming from the virus. These sequences were then mapped to confirm their source.

Concerning the difficulties in including phages in RVDB due to their resemblance to some vectors, a technical question for Pei-Ju Chin enquired about how they manage sequences of animal viruses that also resemble vectors. **Pei-Ju Chin** clarified that these similarities are provided in the annotation sheet. Since the annotation pipeline is now working well, the future inclusion of phages could be discussed further in the AVDTWG subgroup C. Phages may provide a broader view of contamination from bacteria.

A question on the AZ method validation asked which negative and positive controls were used and how raw data are stored in the QC environment. **Bradley Hasson** explained that they use process controls. The method is validated in a modular fashion, with expectations for the assay set at different gates and the process controlled at each gate. They periodically use reagent-only runs in the laboratory for negative controls and investigate any positive signals to identify their source. Regarding data storage, he confirmed that, to date, all the NGS data has been saved. Since 2016, they have accumulated 500 TB of NGS data stored. As a CRO, they are considering different options for data retention. There is also the question of who is responsible for storing the data: the CRO or the sponsor/license holder? For now, they are retaining all the original raw data but are considering keeping only the FASTQ files.

**Marc Eloit** was asked about the standards/reference materials used for the transcriptomics approach. He clarified that they do not mix cells, but RNAs extracted from the cells to be used as controls. These are frozen and used for different runs. For now, each lab needs to make its own materials for validation with its own cells. But if cells are distributed by an external laboratory as frozen RNA, these could also be used as a standard. **Arifa Khan** added that they are developing virus-infected cells characterized in terms of virus genome copy numbers. They have different expression levels and are to be provided as vials for direct use. Expression is characterized before and after vialing to monitor its stability.

**Alice Alston** and **Bradley Hasson** were asked to describe the 32-day TAT process. **Bradley Hasson** answered that, in general, GMP assays involve a lot of processing and paperwork. The TAT starts from date of lab initiation. It takes two to three days to get NGS through the lab onto the sequencer (extraction, QC, etc.). The run can take 19–29 h, depending on the chemistry or system used. Then, the data needs to be migrated and analyzed. Afterward, results undergo secondary qualification and a quality audit. He added that they keep trying to optimize the process and reduce TAT.

A question to **Pei-Ju Chin** enquired about performance comparisons between the new RVDB version and other databases such as GenBank or RefSeq. **Pei-Ju Chin** clarified that benchmark speed and sensitivity performance comparisons were made years ago when RVDB was first released. They did not assess correlations with spiking concentration because this is not the purpose of RVDB. For specific situations, it may be important to follow up with the use of a public domain database, which can retrieve higher sensitivity (more hits). However, the additional hits obtained will most likely be non-viral sequences. A follow-up question continued, asking whether the background noise is also expected to be reduced when using RVDB compared to other databases. **Pei-Ju Chin** answered that in the past IABS conference [7], one participant reported a higher specificity (lower background) when using RVDB, compared to other databases.

A follow-up question to **Bradley Hasson** enquired whether they could get enough nucleic acids from testing reagents only in the negative controls. He confirmed that they use an agnostic amplification technique based on a rolling circular amplification of cDNA retrieved from water.

**Arifa Khan** asked a general question to the CROs on how they plan to manage the increased workload and still meet the expectations. **Bradley Hasson** confirmed that they have expanded their capacity over the past years. Recently, they had a major capacity expansion in their facility, incorporating all advised contamination mitigations. This includes different rooms for library preparation to allow the processing of samples from different backgrounds separately at the same time. This expansion enables large sample processing capacity, but this will have to be continuously evaluated. **Marc Eloit** explained that they have two sites (France and U.S.), and the U.S. lab is a large laboratory with high capacity. Regarding bioinformatics, they have been increasing the speed of calculation without consuming too many resources.

## 4 Session 3: NGS applications

This session was moderated by Michael Wall (Health Canada, Canada) and Blandine de Saint-Vis (Boehringer Ingelheim Health Animal, France).

### 4.1 Non-targeted vs broad-spectrum targeted NGS

**Gibran Horemheb Rubio Quintanares** (PEI, Germany) presented on “Comparison of non-targeted and broad-spectrum targeted NGS for adventitious virus detection”

At PEI, the detection of AVs needs to be assessed in biological products such as vaccines and blood-derived products. To obtain these products, plasma from different donors is pooled into one mixture. Plasma pools may contain plasma from 3000 to 36,000 donors, making sensitivity a crucial factor. The broader use of NGS for AV detection has prompted PEI to gain hands-on experience and design methodological approaches to gain a deeper understanding of NGS capabilities.

A fully unbiased sequence analysis does not exist. Every step of the workflow, from extraction to bioinformatic analysis, will bias the detection of different virus types. For example, the preamplification step strategy may enrich single-strand RNA, double-strand RNA, or DNA [46]. Targeted NGS strategies are generally thought of as a narrow spectrum of very selected viruses targeted with specific primers. The strategy presented here is a broad-spectrum capture system that includes the use of capture probes, VirCapSeq-VERT, developed by Columbia University [47]. This system targets coding sequences from more than 207 taxa from all viruses known or predicted to infect vertebrates. Taxa sharing >85 %–96 % identity were grouped. On average, probes were designed to be ≈ 70–79 nucleotides long but could range from 50 to 120 nucleotides. The newest version of VirCapSeq-VERT has ≈700,000 hybridization probes designed for the regions of homology across viruses. Thus, probes are not designed per virus but per identity group. There are multiple probes for each group.

The sequencing workflow is different for non-targeted and targeted NGS analysis. For non-targeted sequencing, the workflow at PEI starts with a single total nucleic acid extraction. The process is then similar to that described in previous presentations, including pre-treatment with benzonase and other nucleases. Part of the extracted material undergoes cDNA synthesis, and the other part is treated as DNA. The two are then combined to create double-stranded DNA/cDNA. For the targeted approach, named as BLOODVIR System at PEI, no nuclease treatment is applied. The total nucleic acids of the sample are divided in three. Two are treated as RNA but denatured at different temperatures: the usual 75 °C that works for most viruses and a higher temperature (95 °C) that works best for orthoreovirus. The other part is treated as DNA. The three parts are then mixed to start the library. In the final step of library prep, the PCR has only 10–11 cycles to avoid introducing bias. The capture consists of hybridization with the biotinylated probes. After the capture, there is a second 16-cycle amplification. This method is used in the clinical field with up to 48 multiplexed samples. For testing of plasma pools, multiplexing was tested with 24, 12 and 2 samples. It was finally decided to advance with 24 samples after confirming these allowed enough sensitivity and coverage.

For the comparison of the two approaches, WHO reference panel for NGS virus standards were spiked into the plasma pools at viral loads ranging from 10^1^ to 10^6^ genome copies per mL. Sensitivity (LOD) was determined as the lowest viral load allowing viral detection in at least three out of five replicates. The percentage of full genome coverage was also compared. For BLOODVIR System, 17 million reads were expected, versus 100 million reads for the non-targeted approach. Sensitivity was highest for EBV and for PCV (10^1^ for both targeted and non-targeted approaches), and lowest for FeLV (10^3^ for the targeted approach and 10^4^ for the non-targeted approach). However, coverage was not optimal for EBV (<10^3^). An in-house developed 21-virus panel was then used to assess the breadth of detection. All 21 viruses were detected with both approaches.

As described above, the workflow is different in both approaches, but the costs of the wet lab part are similar. However, sequencing costs are lower for the non-targeted approach. The cost saving achieved with VirCapSeq-VERT ranged between 48 % and 54 % compared to the standard metagenomics approach, depending on the chemistry and number of multiplexed samples. With the non-targeted approach sequencing each sample at a depth of 600 or 400 million reads, the cost reduction of using the targeted approach instead can reach 98 %.

An ongoing project is now testing the targeted approach with plasma samples, obtained via collaboration with Mexican blood banks. Samples were pooled into pools of 100 samples and metapools of 1000 samples. In total 895 pools were prepared with samples from 89.500 donors. The bioinformatics pipeline includes steps to convert DNA sequence to protein, compare with viruses, and filter for contiguous regions, depending on genome length. Coverage is also inspected, including number of reads and coverage depth. Preliminary results of 40 pools from 400 donors allowed to identify Pegivirus and Torque teno virus in >90 % of the pools, and HHV-6b in 22 % of the pools. Ten other viruses were identified in 2.5–5 % of the pools. Some of these can be explained in the context of the region where they were identified.

### 4.2 Long-read vs short-read NGS technologies

**Megan Gura** (Regeneron, U.S.A.) presented on “Comparison of long- and short-read sequencing methods for recombinant adeno-associated virus sequence and adventitious agent identification in a GMP environment.”

The field of cell and gene therapy has made many advancements in recent years. Recombinant adeno-associated virus (rAAV) therapies have the potential to bring medicine to previously untreatable illnesses. An example is a case reported in May 2024 by the BBC, where a little girl born deaf had her hearing restored thanks to rAAV treatment of a single-gene defect in the otoferlin gene.

Regeneron's rAAVs are made through the triple-transfection of HEK293-T cells. The rAAV is an encapsidated ssDNA virus that contains a gene of interest (GOI) between two inverted terminal repeats (ITRs). The product's critical quality attributes (CQAs) must be characterized when manufacturing rAAVs. CQAs include sequence identity and surveillance for contaminants such as non-target rAAV GOI sequences or adventitious agents. Two sequencing approaches are available in the Regeneron QC Virology laboratories. Illumina NextSeq 2000 uses sequencing by synthesis, releasing fluorophores as new nucleotides are incorporated into the new strand; it produces hundreds of millions of short reads and is extremely accurate (99 %). Oxford Nanopore Technologies (ONT) PromethION sequences by interpreting the voltage generated by each different nucleotide as the DNA (or RNA) passes through a pore; it generates millions of long reads, and it is very accurate (92–97 %), although less so than Illumina. This study used double-stranded DNA as input in both methods: 50–500 ng for Illumina and 1 μg for ONT. Adaptive sampling is a unique feature available on the PromethION that allows the enrichment or depletion of target sequences. The system rejects any strands not matching the provided reference sequence.

For this experiment, rAAV drug products were digested with DNase before performing a column-based DNA extraction. Illumina and ONT libraries were prepared from the same material and sequenced. Data were analyzed using an in-house developed bioinformatics pipeline.

A first comparison between normal and adaptive sequencing on the PromethION allowed to verify that adaptive sampling yields more reads (both rAAV and non-rAAV) than normal sampling. This was explained by the fact that adaptive sampling works best for long (>1000 bp) reads. Short reads were sequenced before adaptive sampling could reject them. However, adaptive sampling has reduced the number of non-target (non-rAAV) reads and was further selected for the Illumina ONT comparison experiment.

As expected, Illumina sequencing produced far more reads than ONT, while ONT produced far longer reads than Illumina. However, the average read quality (Q-score) was approximately the same. While doing this analysis, the Illumina chemistry was updated (XLEAP-SBS chemistry), drastically increasing read quality. This has pulled Illumina's read quality back into the lead. Illumina sequencing struggled to sequence ITRs, but with a 10x base coverage (and 1000x average coverage) criterion, both technologies achieved 100 % genome coverage. The problem with ITR sequencing is related to different combinations of the two orientations of the remaining portion of the wild-type rAAV genome. While the short Illumina reads cannot capture these combinations, ONT's long reads are ideal for sequencing these regions.

The rAAV product has a high titer (10^13^ viral copies/mL). Thus, even minuscule cross-contamination can be problematic. To test for cross-contamination, decreasing amounts of one rAAV (REG v5) were spiked into the other (REG v4) down to 0.1 % of the sample volume. A no-spike control was included. Both technologies were able to detect REG v5 uniquely mapped reads at all spike levels, although Illumina coverage data were slightly higher than ONT. After applying the coverage criteria, both technologies had the same LOD.

Detection of rAAV variants is important for drug product potency specifications. A double-stranded DNA construct was created to test the detection of sequence variants with mutations, insertions, and deletions added. These constructs were then sequenced with both platforms. Illumina sequencing successfully detected all variants, and ONT sequencing detected all variants in all constructs except for one (a multi-nucleotide variant in the 3’ end); however, this run had an unusually low read coverage.

Illumina sequencing was overall more cost-effective for this purpose than ONT. The caveat is that the experiment used multiplexing (up to eight samples) on Illumina and not on the PromethION. Manufacturers recommend multiplexing up to 30 samples on Illumina and up to 24 samples on PromethION. In that case, the cost reagents per run would be lower on the PromethION than in Illumina.

Both instruments were found to have their strengths. The team continues to experiment on both platforms and will try to incorporate the PromethION in future investigational testing or other methods.

### 4.3 Release testing vs. investigational follow-up

**Carine Logvinoff** (Sanofi, France) and **Song Sun** (Sanofi, Canada) presented on “Selecting and developing approaches for adventitious virus detection by HTS, contrasting release testing with investigational follow-up”

The traditional testing package for AV includes non-specific (such as *in vivo* assays, cell culture tests, infectivity assays, and electron microscopy) and specific (such as 9CFR for bovine and porcine viruses, *in vivo* MAP/RAP/HAP tests, or PCR) test methods. Each method targets different viral characteristics, and none of them can detect all viruses at once. Limitations of these methods have been extensively described in previous sessions (see section 2.1). Non-specific test methods need a match between the model and the virus to allow its detection [30], and they must be complemented with a specific test. Specific tests need to select which virus to test for and consider the viral variants. The conventional testing package could miss viral contaminants. An example was the already-mentioned discovery of PCV1 in a rotavirus vaccine in 2010 [3]. This work highlighted the potential of HTS to identify known and unknown AV, replacing *in vivo* assays and all the specific test methods.

*In vivo* tests are typically performed on cell banks and viral seed lots. Sanofi's goal was to replace these tests with one approach that can fit all matrices (cells and harvest with more or less cellular components). Since the new vaccines are produced with animal-free materials, they selected a pipeline capable of detecting any viral nucleic acids. The new method was assessed by characterizing its specificity, breadth of detection, and sensitivity, using a panel of 16 model viral stocks developed by the National Institutes of Health (NIH) [48]. The 16 NIH viruses represent potential contaminants that could be introduced during vaccine production and include human and animal viruses from a variety of families, RNA and DNA genomes, and enveloped and non-enveloped viruses [30]. The NIH protocol was used to produce and characterize the viral stocks, which allowed linking the HTS data with the published *in vivo* data. Using viral stocks as representative spiking materials in the detection pipeline allowed documenting the breadth of detection and sensitivity of the HTS pipeline. The data package supports the substitution of an *in vivo* test by HTS for AV detection test, as recommended within Ph.Eur. 5.2.14. HTS allowed streamlining the AV testing package and strengthened product viral safety.

Sanofi's testing pipeline follows a typical viromics and genomics approach. It favors detecting encapsidated viral nucleic acids but also detects those non-encapsidated to a certain extent, via removing host nucleic acids during pre-extraction treatment to improve the sensitivity of detection. Pseudoviral particles for DNA and RNA viruses are used as internal controls and added to the sample matrix before pre-extraction treatments. These two internal controls assess the performance of the different steps of the test, including pre-treatment, nucleic acid extraction, library preparation, sequencing, and bioinformatics analysis. General controls include measures to avoid cross-contamination (segregate lab rooms with dedicated biosafety cabinets for nucleic acid extraction and library preparation and unidirectional workflow; dual-indexed sequencing library), quantification of input DNA for library preparation, assessment of the sequencing library (size and concentration), and read quality for sequencing run (minimum % Bases Q ≥ 30). To ensure that the HTS workflow is robust across different sample types, matrix-specific validity criteria are established for total output per sample (number of reads) and for the recovery of the internal controls (number of reads detected as the two internal controls).

The selected spiking materials for method development and validation are NIH and WHO reference viruses, which represent viral diversity in terms of structure, nucleic acid type, genome size, virus morphology, and chemical resistance. These reference model virus stocks are characterized for genome copy number, infectious titer, viral genome sequence(s) including any variants, and any additional expected background signals (e.g., from production substrates).

Before validation, matrices are assessed using representative samples to evaluate the optional pre-extraction steps and potential procedure modifications for method improvement (e.g., adding a new step, replacing a reagent kit, or upgrading bioinformatics software) and to determine target LOD and specificity with the model viruses. Matrix assessment also allows for demonstrating assay robustness, determining validity criteria for internal controls, and identifying false-positive generating signals from matrix or testing reagents.

The validation execution should follow a pre-approved validation plan, which details the validation characteristics (LOD and specificity), the study design, the qualification of internal controls as critical reagents, and the validity and acceptance criteria.

As a generic validation, Sanofi's HTS method was validated using a Viral Crude Harvest Matrix by demonstrating sensitivity (LOD) using 16 NIH viruses spiked in at 10^4^ copies/mL and specificity (breadth of detection) using a negative control extracted and sequenced in parallel. The two internal controls were added to the matrix to assess extraction efficiency and viral nucleic acid recovery. This validation study included two LOD runs and one specificity run, with LOD runs conducted by two independent analysts over two days to ensure robustness.

For any new matrix, one must first assess whether an equivalent matrix has been previously validated. If not, the method development and generic validation described above are followed. If yes, the potential impact on the LOD or specificity for the new matrix should be assessed considering attributes such as antigens expressed, manufacturing process, or freezing medium. If there is an impact, the process returns to method development. If not, at least one run is performed to verify LOD and to confirm the validity criteria for internal controls. After this verification, testing is done using the validity criteria used for the equivalent matrix.

When a signal is detected, the process advances to follow-up investigation. This is built based on the identified signal(s) with dedicated tools to conclude on the replicative status of this potential viral contaminant. Different tools can be used for investigational follow-up. One tool under development is a generic orthogonal PCR method that leverages the sequencing data to design primers/probes. Sensitivity can be estimated using synthetic templates, and multiple samples can be tested in parallel with short TAT. Another potential tool is ONT for long reads, which allows for assessing genome integrity. This technology can sequence native nucleic acids without amplification and analyze real-time data, but it requires a high yield of input material to obtain quality results. Finally, a stranded RNA-seq approach can be used to detect viral RNA with directionality and determine viral replication based on the type of viral nucleic acid. However, this method is matrix-specific, and transcripts may be degraded in some matrices. It also requires a high yield of input RNA.

In conclusion, the detection of (mainly encapsidated) viral nucleic acid was selected for the release test. This allowed to have comprehensive validation packages using well-characterized representative spiked-in viruses (NIH & WHO reference standard) in cell banks or harvest matrices. The LOD observed might be underestimated, as a true contaminant would present additional viral nucleic acid (transcript, replicative intermediate, etc.) not spiked in with the viral stocks used. Follow-up investigations are needed to conclude the replicative status of the potential viral contaminant. These additional tools do not require the representative validation material needed for the release test.

### 4.4 AI prediction of novel viruses

**Martin Machyna** (PEI, Germany) presented on “BLOODVIR – Surveillance system for novel viruses based on NGS and artificial intelligence (AI)”

Viral contaminants are found in different types of biological materials, including in blood donations. In Germany, 6.5 million blood donations are collected per year. Their active screening via a PCR-based method has helped contain transmissions to 11 in 4 years (2016–2020). However, this screening targets a limited number of viruses (HBV, HCV, HEV, HIV, Parvovirus B19). PEI has been developing an NGS-based surveillance system to allow for the identification of known viruses and the detection of novel viruses. This system — BLOODVIR — includes an in-house data analysis pipeline incorporating ML.

The BLOODVIR analysis pipeline is implemented in Snakemake and can be run locally or on a high-performance computing cluster. Reads are classified against a reduced version of RVDB, and novel virus predictions are achieved with a trained ML model. Analysis results are summarized in an interactive dashboard. The pipeline is designed to use any Illumina sequencing approach. This includes non-targeted metagenomics, which is agnostic to virus type or genomic sequence and has limited virus material enrichment except for the host sequence depletion step (nuclease treatment), or for targeted virus enrichment approaches, such as VirCapSeq-VERT (section 4.1), which enables sequencing of genomes with as little as 75 % sequence identity.

For BLOODVIR, the data analysis approach starts by removing any residual contaminating sequences that align with genomes from the host (human), mouse, bovine, or bacteria. Sequences that pass are trimmed and quality-controlled so that only high-quality reads pass to the next step. Sequences are then classified into species. Two options exist for sequence classification: alignment-based (e.g., MiCoP [49], TaxMaps [50]), or K-mer-based (e.g. Kraken2 [51], Centrifuge [52]). These methods were tested with an *in silico* dataset of a simulated mixture of virus sequences, human, and a random sequence at different concentrations. All methods worked well for a low mutation rate, but MiCoP provided the best classification accuracy across a range of editing distances. RVDB was used as a viral genome database, but an initial investigation has shown that this database has an unbalanced distribution of species, with an overrepresentation of some species over others. Thus, a custom filtering step was applied to remove genomes with a MinHash score <0.15 and sequences <1000 nucleotides. RefSeq records were retained. This substantially reduces the database and makes it more balanced, with better performance for MiCoP classification. A second classifier (Kaiju) based on the protein database was introduced. BLOODVIR could reproducibly detect 10 viral copies/mL. Sequence classification was then used to calculate genome coverage and estimate species abundance, which were combined into an interactive dashboard report.

Unclassified reads are assembled into longer contigs to predict whether they may originate from a novel virus using a ML model. The model is trained using sequences from RVDB fragmented into smaller sequences. Two types of ML architectures were considered: convolutional neural network with random forest classification, which considers sequences as images and nucleotides as pixels (e.g., Ref. [53]); and a transformer model that considers the sequence as a language with four letters (e.g., Ref. [54]). To be selected, ML architectures had to be published recently, have a publicly available code, and be well maintained. Five architectures were selected and tested for *de novo* virus prediction using a leave-one-out strategy. This consists of training the model with non-viral sequences and viral sequences dataset where genomes from one virus family was removed. Each architecture was trained ten times, each time removing a different virus family. Then, the families left out were provided as contigs for viral origin prediction. The parameters obtained for evaluation were sensitivity, precision, and the F1 score. Results of the assessment showed that, while good sensitivity is obtained across different sequence lengths, the accuracy of novel virus prediction decreases as sequences get shorter. The best-performing architecture was VirHunter, followed by Deep6. VirHunter was also the best-performing architecture when testing with left-out classes instead of families. Thus, VirHunter was selected for implementation in the pipeline. Results from the viral prediction ML model are also included in the report dashboard.

In conclusion, the BLOODVIR pipeline is implemented in Snakemake for reproducible execution. Sequences were classified with MiCoP, which is more accurate than K-mer-based solutions. The pipeline detects viruses at an LOD of 10 copies/mL, and novel viruses are predicted using a custom-trained VirHunter ML model.

### 4.5 Panel discussion: session 3

The session closed with a panel discussion moderated by **Michael Wall** and **Blandine de Saint-Vis** including all the session speakers. **Gibran Rubio Quintanares** was asked how human endogenous retrovirus HERV-K is distinguished from cellular sequences, which also contain this provirus. He clarified that this was included in the first panel but was removed from the most recent versions precisely because it cannot be distinguished from human sequences. After a follow-up question, he added that VirCapSeq-VERT is not aimed at detecting endogenous retroviruses.

**Martin Machyna** was asked how many contigs detected with the new approach were also detectable by BLAST. He replied that more tests are still needed, but in some cases, the contigs were detected by BLAST as originating from a virus. However, many false positives still exist, as the model still requires fine-tuning. Further filtering steps will need to be added, especially for short contigs.

**Gibran Rubio Quintanares** was asked, regarding the criterion of three replicates out of five for detectability, how this would be implemented in a GMP environment or a clinical trial. The replica that missed the detection could be the one with a contamination. He agreed that these thresholds still need to be defined, as this was a first test. A statistical approach where a Poisson-based distribution and statistical mean are defined was suggested (from the audience) to calculate the probability of detection. The broader the dilution steps and the fewer replicates, the less accurate the determination of the LOD. The Ph.Eur. recommends three replicates and tenfold dilution steps, although this does not allow for the achievement of an accurate LOD. A more accurate determination would require more replicates.

A question for **Song Sun** was related to the internal process control for RNA and DNA. Would it be possible for the internal control for RNA to become a DNA control after reverse transcription? He clarified that the RNA control controls the reverse transcription, while the DNA control mimics DNA viruses. The aim is to capture any potential impact from the packaging of these nucleic acids. Having both allows for a risk assessment evaluation. Furthermore, accumulated data on the spiked-in viruses have shown that the nuclease treatment does not impact DNA and RNA viruses equally.

**Martin Machyna** was asked what kind of error model was included in the *in silico* generation. Does it consider distinct types of mutation rates or only single nucleotide polymorphisms? He replied that only substitutions were considered. Insertions/deletions could be included, but they would affect the K-mer models even more than substitutions.

**Martin Machyna** was suggested to consider retaining some classified reads to help contextualize the unclassified reads. He added that, for now, only unclassified were retained. However, there are cases where some reads appear misclassified, and these could potentially be pooled into the unclassified pool. **Arifa Khan** added that although vectors have been annotated, RVDB is still working on the annotation of endogenous retroviruses. Thus, training the model with these sequences may include some non-viral sequences. This may explain why the accuracy is not as high as expected. **Dr. Machyna** clarified that annotated endogenous retroviruses were removed from the training dataset, but those not annotated might still be included and cause problems. More filtering may be necessary.

Concerning the model's training and F1 scores, **Martin Machyna** was asked to clarify if the scores presented were for the fragments for the full pipeline. He clarified that these scores were not yet for the whole pipeline; this work is still pending. In response to a follow-up question, he added that transformer models are unaware of open reading frames and codons.

The panelists were enquired about their experience with the nuclease treatment. **Song Sun** explained that they used the 16 NIH viruses and the WHO reference panel as spiking material. They tested them both with and without nuclease (benzonase) treatment. The recovery of viral material always improved with the nuclease treatment, but the impact depends on the matrix. For simple matrices, the nuclease will most likely impact the virus, but for matrices with a heavy host background, this may protect the viruses to some extent. **Arifa Khan** added that this was a moderate nuclease treatment. Stronger treatments were observed to affect all the RNA viruses but not the DNA viruses. The nuclease needs to be tested with the virus as the effect is virus-specific. **Song Sun** added that there is still a substantial fraction of host sequences after the moderate nuclease treatment. **Gibran Rubio Quintanares** noted that freezing and thawing plasma samples substantially decreased pegivirus protection against nucleases. Using a combined harsh benzonase, DNase, and RNase treatment helped to reduce the background but reduced bunyavirus signals to some extent. This depended on the initial titer.

**Gibran Rubio Quintanares** was asked to comment on protocol changes planned for implementing the technology to cell bank testing. Dr. Quintanares clarified that they had tested the panel with a background of adenovirus at 10^9^ copies/mL, and the results were similar, with a slight reduction in sensitivity. Since probes are designed to capture only the viral sequence, the matrix does not affect this method. They plan to test it and modify it as needed. In theory, this should not suffer interference because the probes are specific for the viral sequence and added in excess to avoid competition. Sample multiplexing also needs to be adjusted to optimize the method. Reducing the number of samples per multiplex will increase sensitivity. A follow up question asked if viral genome size was considered when designing the probes. **Gibran Rubio Quintanares** replied that the space between probes is 25–50 nucleotides long, so longer viruses will have more probes. In the second version of the panel, probes were adapted to balance between long and short virus genomes and achieve a more homogeneous panel.

**Martin Machyna** clarified that the ML model used the clustered RVDB as the basis for further filtering. In reply to a question about not detecting RSV with the original RVDB, he postulated that this could be due to too many similar sequences. Reads then align to multiple sequences (multi-mapping), and the pipeline cannot distinguish which genome to assign them to. These sequences are then tossed out because the pipeline cannot properly assign them. **Gibran Rubio Quintanares** highlighted that the reduction of the database needs to be customized based on the needs.

**Megan Gura** was asked, regarding the adaptive sample, which type of size-based filtering was performed on the human and *E. coli* sequences. She noted that the analysis pipeline was developed by a bioinformatics team. The sequencer was set to filter for reads >20 base pairs (bp) during the run. The bioinformatics pipeline filters for reads >150 bp roughly. For rAAVs specifically, long reads >1000 base pairs are more informative because they cover the full genome. However, it might be useful to keep shorter reads (>500 bp) if they are good quality.

**Megan Gura** was further asked if they characterize small sequence variants as QC. She clarified that the goal of the experiment is to ensure 100 % match to the reference sequence to ensure the rAAV sequence is exactly the one expected for the treatment. For the variant construct, they plan to do further experiments to understand the LOD differences between platforms, and GOI and ITR, and establish an overall LOD.

A comment from the audience shared that adaptive sampling did not work well for AV detection in their experience. From an enrichment standpoint, reads were too small for the system to decide whether the sequence was viral or not, and by the time it decided, it had already passed through the pore. **Megan Gura** was further asked whether statistics were collected on the rejected reads. She clarified that these are available but have not been checked. She added that there is a difference in the analysis for AV and for rAAVs. Uniform and long reads are expected for rAAVs, which makes them more suitable for adaptive sampling. They have also tried depleting against the host background, which could be helpful for AV detection.

A general question from the audience, concerning the short-vs. long-read comparison, asked at what point is the quality good enough? From an adventitious agent detection perspective, does the quality increase matter (from a certain point) versus the possibility of getting very long reads? **Arifa Khan** viewed this as detection versus characterization. For AV detection, the crucial point that needs to be accurate is detection. For characterization, the full virus genome sequence is needed, and long reads are helpful when combined with the Illumina data. **Megan Gura** added that better quality is more important than long reads when detecting sequence variants.

A comment from the audience noted that Nanopore sequencing might have problems with secondary structures formed on the exit side of the pore, which could influence the speed of the rest of the DNA sequence. This could be reflected in Megan Gura's coverage plots, where ITRs have higher coverage than the rest of the sequence. A suggestion would be to separate the sequence into two regions to be sequenced separately: the ITRs and the GOI. **Megan Gura** replied that the higher coverage at the ITRs may be related to the ligation step and the adapters at the end of the rAAV sequence. Maybe reads are shorn during nucleic acid extraction in library preparation resulting in shorter sequences. However, they have not tried separate sequencing of the ITRs or the GOI.

After being requested for further details on the adaptive sampling, **Megan Gura** clarified that they use an onboard algorithm and sequence at super high accuracy basecalling. They have not tried the fast 240, which could help make the system decide at shorter sequences. They use a FASTA file as reference, but a bed file could help take faster decisions.

Regarding Sanofi's validation, **Song Sun** was asked if there were differences between virus families and between matrices. He clarified that there was a matrix effect on the sensitivity. Regarding virus families, in matrices where the background was strong, the improvement achieved with nuclease treatment was stronger for RNA viruses (>100-fold increase) than for DNA viruses (≈10-fold increase). This could be related to an interference of the host background material with converting RNA to cDNA. This is why new matrices need to be assessed to evaluate the need to optimize some steps.

**Martin Machyna** was asked a question regarding the filtering step: What is considered a separate/different genomic region? He explained that sometimes the alignment could be heterogeneous across the genome, with only some spots well covered. These spots are combined to get a minimum of three non-adjacent regions or at least 1000 nucleotides of combined genome coverage to be considered as positive hit. These filters are empirical and aim to find a correct threshold for what should be considered real. This criterion is based on the one used for PCR, where at least three different regions of the genome need to be obtained to confirm virus presence. The limits in alignment percentage or score also need to be defined. The current strategy is to do a standard BWA alignment (with no threshold) and direct anything that does not align to the ML pipeline.

A question from the audience addressed the issues of multi-mapping in databases with redundant sequences. Would it be possible to reduce sequences so that they capture the variance in genomes? Variants shared between multiple species from the same family could be redundant. For the VirCapSeq-VERT pipeline, reads are not assembled because blood plasma pools contain sequences from hundreds to thousands of individuals, which would create assembled chimeras. In this case, it was preferable to use single unassembled reads. Multi-mappers occur with different approaches, such as BLAST and K-mer-based methods. The strength of MiCoP is that it assigns multi-mapping reads in a probabilistic way based on the percentage of unique mapping reads.

**Arifa Khan** clarified that the purpose of the unclustered RVDB was to retain sequence diversity to help detect distantly related sequences, while the purpose of the clustered RVDB was to reduce the redundancy of 98 % of the sequences. However, both databases can be customized to adapt to the user's questions and pipelines. Pei-Ju Chin further appealed to the audience to join AVDTWG and foster these discussions within the database subgroup C. NCBI is now also joining the working group discussions, as they realized there was a problem with the SARS-CoV-2 sequences. RVDB aims to retain the completeness of the database, and it is up to the users to further reduce it or not, depending on their risk assessment and pipeline.

## 5 Session 4: strategies for optimization of NGS virus detection and follow-up of NGS signal

This session was moderated by Siemon Ng (Notch Therapeutics, Canada) and Marie Murphy (Eli Lilly & Co., Ireland).

### 5.1 Alignment-free adventitious virus detection using NGS

**Tom J.B. de Man** (Bioreliance, Merck, U.S.A.) presented on “Rapid alignment-free detection of adventitious agents using NGS”

Metagenomics NGS (mNGS) starts with extracting nucleic acids from a sample and is culture independent. mNGS yields many sequences that need to be classified into their taxonomic source. The growing volume of mNGS data has driven the need to develop new algorithms and bioinformatic pipelines to analyze this type of data. Algorithms evolved from BLAST to BWA, then Bowtie2, VirFinder, Kraken2, DeepMicrobes, and Stat, correlating with an increasing size of mNGS datasets and reference databases. At Merck, the development of new bioinformatic tools for AV testing is largely associated with the use of new sequencing platforms with higher throughput. The new metagenomic classifier, presented during this talk, is associated with an Illumina NextSeq2000 sequencing platform that produces an average of 400 million reads per run.

The new metagenomics classifier was deemed necessary due to constraints associated with large reference databases, lengthy database build-time, redundant results, computational intensity, and limitation to small genomes. The revised pipeline presented here uses exact K-mer matching. It uses a smaller database and avoids duplicate results. Decisions are binary for study management, and it allows for analyzing large genomes from bacteria and fungi.

A K-mer is a subsequence of length k derived from a longer nucleic acid or protein sequence. Each K-mer is derived from the parent sequence by moving one nucleotide to the right. K-mer-based algorithms are fast because they use exact matching rather than inexact sequence alignment, reducing all decisions to binary decisions. The new pipeline also uses the lowest common ancestor (LCA) for data compression. With this strategy, K-mers aligning to more than one species will be assigned at the taxon level of their LCA. This allows assigning K-mers to a unique taxon. Additional K-mers may help inform on lower taxa.

Platform validation used data published elsewhere [36]. Three representative RNA viruses (REO1, FeLV, and RSV) were spiked into four cell lines commonly used in biomanufacturing (HeLa, CHO, Vero, and Sf9) at three spike levels: 10^5^, 10^4^, 10^3^ VGC per 10^6^ cells. An unspiked control was also added. For the new K-mer algorithm validation, LOD was defined as the lowest dilution in which the virus was detected in at least two out of three replicates. Data qualification was also performed by following up on positive hits.

The mNGS assay's specificity was evaluated by analysis of the unspiked cells and virus-spiked cells at the highest spike level of 10^5^ VGC, with no viral spike sequences detected in the unspiked controls. The LOD was 10^3^ VGC/10^6^ cells for RSV and 10^4^ VGC/10^6^ cells for FeLV and for REO1. The overall LOD of the assay was determined to be 10^4^ VGC/10^6^ cells across all four cell lines tested. In a few replicates, REO1 was not detected at the species level. Taxon-specific K-mers have a higher weight in the classification than conserved sequences. This explains why, at the species level, fewer reads were detected with the K-mer algorithm than with the read mapping algorithm. Furthermore, since K-mers are short sequences, they are less specific than full reads.

During data qualification, each positive result of a virus, regardless of taxonomic level, potentially infectious to the test sample and/or capable of harming human health was confirmed through additional comparative analyses. The K-mer results contain read classifications but not their aligned positions in the genomes. Although K-mer matching can be less specific than read mapping, subsequent data qualification allowed for the confirmation of K-mer sequence placement within the (viral) taxonomic tree, resulting in enhanced taxonomic resolution. This approach enabled the classification of REO1 results at the correct species level.

Genomic reference database curation is key for mNGS analyses. Metagenome-assembled genomes (MAGs) are noisy representations of a viral genome, and they represent 1.8 % of the current reference database. They cause problems with the analyses because they are often mislabeled and of poor quality. MAGs are generated via numerous strategies and may originate from a pool of data from hundreds of samples using different NGS platforms, extraction and library preparation methods, and quality control criteria. Thus, MAGs have been removed from the analysis for now, but the topic was suggested for further discussion within AVDTWG subgroup C.

In conclusion, employing a metagenomics classifier (K-mer) algorithm has future-proofed mNGS adventitious agent testing operations. The overall LOD of the assay was determined to be 10^4^ VGC per 10^6^ cells across four cell lines tested. Adventitious agent testing data qualification increased taxonomic resolution and relevancy of the K-mer results.

### 5.2 Metagenomic analysis to complement viral risk assessment

**Vanessa V. Sarathy** (MSD, U.S.A.) presented on “Viral metagenomic analysis to complement the viral risk assessment and adventitious agent testing of live virus vaccines”

Viral metagenomics uses NGS to identify contaminating viruses in biological products. As described in previous presentations, NGS testing can supplement gaps in traditional adventitious agent testing of live virus vaccines or as part of an early de-risking strategy for clinical studies. MSD applies a risk assessment before starting a metagenomic analysis study. This helps determine the types of study samples, the test articles, the controls, and the type of analysis (viromics versus transcriptomics). Specificity and sensitivity are determined by spiking with the CBER NGS Virus Reagents to determine recovery. Synthetic readsets containing representative model virus sequences are used to evaluate the bioinformatic analysis aspect of the testing. NGS is performed using the Illumina short-read workflow (paired-end, 2x150 bases). Results from bioinformatic analysis are followed up with manual analyses.

The short-read alignment workflow starts with sample preparation, extraction and sequencing. Reads are then processed for quality. Read sets from samples with the vaccine virus are stringently aligned to the vaccine virus reference sequence to subtract those from the subsequent analysis. The resulting reads are then aligned to a curated version of the RVDB that has been appended with the vaccine virus sequence to control for cross-contamination or minor variation from the vaccine virus sequence. The best hits within taxa are reviewed across the study samples, and the putative positives are counter-screened. Bioinformatically-confirmed hits proceed for further investigation.

Bioinformatic analysis for regulatory filings uses a High-performance Integrated Virtual Environment (HIVE). It is maintained by the experts at George Washington University, and it has well-known and published tools available in public instances, such as the HIVE Hexagon HTS read aligner and the HIVE Heptagon alignment profiler. It can also generate BioCompute objects for pipeline communication. The HIVE workflow is highly manual, including input of read sets and references for alignments. Next, command line support for taxonomic processing is followed by the use of complex worksheets and pivot tables. The process allows for the determination of the accession numbers for taxa with the maximum coverage and the maximum total number of alignments.

To automate and streamline this data analysis process for GMP or release testing, MSD has internally developed ViruScreen, a bioinformatic analysis application for viral metagenomic analysis. ViruScreen is user-friendly, works within the company firewall, and allows for customizable workflows for GMP or non-GMP environments. The application generates a downloadable report. Multiple workflows are available in ViruScreen for Illumina paired-end reads. This presentation addressed database curation, short-read alignment, and *de novo* assembly alignment.

The clustered RVDB is curated to reduce positive hits that could arise from host sequences in bulk vaccine virus harvests. A process described elsewhere is used [55]. Sequences are split into pseudo-reads, taxonomically classified, and aligned to custom databases of humans, bacteria, vectors, etc. Dustmasker is used for low-complexity regions. Undesired nucleotides are converted to “N” to retain the same number of records.

The automated stringent short-read alignment step starts from FastQ files. After the stringent alignment to the vaccine virus sequence, sequences are then aligned to the curated RVDB with Bowtie2. The workflow includes several custom Python scripts, yielding tables with the best accession hits for each taxon (species, genus, family). Coverage maps are also generated.

A case study to benchmark ViruScreen GMP against HIVE was presented. The purpose was to analyze datasets representing different types of data and sample preparations to inform on the sensitivity and specificity across HIVE and ViruScreen workflows. Research datasets representing *in silico* spike data with millions of reads of background media were spiked with known virus amounts or virus sequences for comparison. The analysis involved short-read alignments of different spiked datasets to the clustered RVDB v.20 with added curation. HIVE alignments were performed with Hexagon, and ViruScreen alignments were performed with Bowtie2. Results determined that the recovery of the spiked viruses/virus sequences by maximum total hits and coverage length was similar with both workflows.

A larger study was then designed with the live dengue vaccine. V181 is an investigational live attenuated quadrivalent dengue vaccine produced in Vero cells. Non-GMP samples of the V181 dengue 3 vaccine and controls were used for a spike recovery study using the CBER NGS Virus Reagents (spike levels 10^3^, 10^4^, and 10^5^ VGC/mL). Unspiked samples were used to generate research results and understand the workflows. Read sets followed a short-read alignment to a curated, clustered RVDB v.25 using HIVE and ViruScreen. *De novo* assembly alignments were performed in ViruScreen using Megahit and Magic-BLAST to compare with short-read alignment. Results show that overall, in the short-read alignment across unspiked samples, HIVE returned the most hits. This could be partially due to the lack of paired-read filtering in the HIVE workflow. In ViruScreen, the short-read alignment led to a higher number of putative hits to counter-screen than the *de novo* alignment workflow.

For counter-screening, two examples were provided. In the first example, coverage plots showed high coverage in specific sequence regions of HIV-1, but large regions were not covered. Different BLASTn and BLASTx alignments demonstrated the best match to non-viral accessions, and there was no unique coverage for the identified virus (HIV-1). Thus, this hit was not bioinformatically confirmed. The second example was provided to simulate counter-screening of a true positive from a sample that was spiked with the FDA virus panel, and thus contained virus sequences; coverage maps showed coverage across the whole sequence of pangolin circovirus. BLASTx and BLASTn alignments demonstrated the best match to PCV1 replicase and high match scores to several circovirus species. This hit was confirmed as a true positive, the PCV1 spiked into the samples.

In conclusion, Viral Metagenomic analysis studies have been undertaken to supplement the virus risk assessment for select live virus vaccines and cell banks. HIVE bioinformatic analyses have been successfully filed as characterization studies, and an internal application for customized workflows (ViruScreen) was created to streamline bioinformatic analysis for a potential future release of GMP materials. Comparison across workflows allowed benchmark bioinformatic methods. Large datasets from the dengue live vaccine samples were generated to complement the initial comparisons between HIVE and ViruScreen, which exhibited similar performance in spiked virus recovery. Additionally, end-to-end workflow testing led to identifying successes and opportunities to leverage in studies for filing advanced virus detection methods for biological products. Further automation of largely manual analysis, such as counter-screening and comparative analysis in ViruScreen, will facilitate future applicability for product-specific release or GMP assays.

### 5.3 Bioinformatics pipeline for adventitious agent testing

**Robert L. Charlebois** (Sanofi, Canada) presented on “Evolution of a bioinformatics pipeline for adventitious agent detection”

Bioinformatic analysis is the final step in the NGS process. NGS assays and bioinformatics platforms are modular, allowing for the upgrade of specific system steps. The potential steps in a bioinformatic pipeline for NGS data analysis have been well described by Lambert et al. [56]. PhyloID is a bioinformatics pipeline developed to identify putative adventitious agents (with a focus on viruses) in a sample (cell bank, seed lot, viral harvest, etc.) from a dataset of NGS-generated nucleotide sequences. The assay (wet lab and bioinformatic pipeline) was first developed in 2010, as a response to the “circovirus crisis” [3] and GMP-validated in 2017 [48]. The presentation described the development of the PhyloID pipeline.

PhyloID v1 had a Linux command-line interface. The core strategy was based on building phylogenomic distance matrices from a trusted sequence repository [57]. RVDB was still starting, so RefSeq was used as the reference sequence source. To reduce the size of the data for computational efficiency, data were compressed with *de novo* assembly, and BLASTn was used for screening and counter-screening. The pipeline used a phylogenomics approach to identify sequences. At that time, the pipeline provided a good signal-to-noise ratio, mitigating the fears of opening Pandora's box. However, there were several challenges: building the reference genome collection was slow and laborious; the *de novo* assembly could lead to chimeric sequences and misidentification; the analysis pipeline was slow; and signals needed to be followed up manually.

PhyloID v2.0 (2022–2025) was a major upgrade. New modules were added, and others were improved. The cloud implementation allowed for parallel processing. New quality filters avoided misclassifications, and host filtering cleanly subtracted the host, reducing the size of the dataset. Ordinary deduplication avoided chimeric sequences. K-mer-based phylogenomics automated reference curation, making it 1000x faster. Automated follow-up of signals and a semi-automated decision tree to find actionable signals were introduced. A better, web-based user interface was available, with automated reporting. This version was much faster and more automated, but it was still slow and expensive for bacteria, and it provided incomplete support for retrovirus detection.

PhyloID v2.1 is currently being validated. It uses a position-aware K-mer approach to find viruses and/or bacteria, which is sensitive, specific, and fast. It continues using the v2.0 pipeline to resolve non-obvious viral signals. It incorporates a curated and more complete virus database, including RefSeq and RVDB, and brings retrovirus detection into scope. The decision tree is now more automated. Information can now be output as JSON files for better interoperability. The interface is simpler, with better reports. This version is faster, more affordable, and more automated, including bacteria and retroviruses in the scope. Challenges are now related to the need for better models for background signals (work in progress) and incomplete support for truly novel viruses.

In summary, PhyloID v1 met expected viral safety commitments. It mitigated the risks of missing something, with extra caution regarding false positives. Extensive development was needed before validation to inform the design of decision trees, including discussions (internal, and within AVDTWG) and stakeholder education. PhyloID v2.0 could get the job done more efficiently to better meet project timelines. As HTS-based AV testing gained internal acceptance and adoption, PhyloID v2.0's scalability and automation accommodated that greater demand without increasing specialist workload. It also leveraged past experience to understand what impacts risk for both false negatives and false positives, informing the design of new application modules. Automated decision trees placed the software at the front line. V2.1 is now expanding the scope and setting up for the future. It addresses recognized gaps, in consideration of future testing package streamlining to include retroviruses and microorganisms. Its performance was further improved. This version sets up data structures for later PhyloID versions to better manage background signals.

### 5.4 Panel discussion: session 4

The session closed with a panel discussion moderated by **Siemon Ng** and **Marie Murphy** involving all the speakers. A comment for **Tom de Man** noted that their strategy identified REO virus in the qualification test only because the virus was known to be present. What would be the strategy for detection when the virus’ presence is unknown? Dr. de Man explained that the first step is analyzing with K-mers. The subsequent qualification uses a separate read-mapping approach. Here, no pre-knowledge is required because all the classified viral reads from the K-mer analyses, irrespective of their LCA taxonomy, are compared against a comprehensive viral and cell-line database. This may result in a change in the taxonomic group.

**Robert Charlebois** was asked if there are risks in missing taxonomies by using RefSeq to iterate RVDB since NCBI might not include all taxonomies. He explained that the approach consists of super-sampling RVDB and using K-mers to identify unmatched sequences against RefSeq. The worst-matching member of that species in RVDB is pulled from GenBank and added to the collection multiple times until a full set of representative sequences has been added. This should capture all the taxa that are not represented in RefSeq.

**Robert Charlebois** was asked about improvement metrics for PhyloID. He clarified that the reference genome collection time decreased from seven months on a cluster in v1 to two days on a single node in v2.0. Sample analysis time decreased from one month to a week, even with the database's growing size. Certain steps of the process are serial which prevents higher time savings. Nevertheless, the process is 20–25 % faster with v2.1, and it is less expensive than in previous versions. Sensitivity is comparable, but it now has a broader scope. The K-mer approach is protein-based, so fully intergenic reads will not be detected, which results in a few percent decrease in sensitivity.

**Vanessa Sarathy** was asked about the plans to move forward with the ViruScreen versus the HIVE approach. She expects to continue using HIVE in the research space but will continue to invest in an internal application for GMP purposes. This is expected to grow with the implementation of the ICH Q5A(R2) and the expansion of the types of samples and modalities of the work. The workflow will thus diversify. In response to a follow-up question Dr. Sarathy clarified that HIVE can be used free of charge, however, has a collaborative agreement in place, which compensates for bioinformatic expertise and support costs associated with the use of proprietary data and security assurance.

**Tom de Man** was asked how K-mer classification models deal with potential mutations seen in read sequences. He clarified that in the qualification step, they use read mapping for all viral results, irrespective of taxonomy level (from viral kingdom to strain), which is more flexible for detecting mutations. Unclassified reads can be screened against a database with ≈40,000 sequences from different viral species and strains. When something new does not match the database, it must be investigated.

Regarding the plans to include retroviruses in the scope of PhyloID, **Robert Charlebois** was asked how it would be possible to distinguish endogenous from exogenous retroviruses. He answered that the database can detect the signals, but this will involve some wet lab work, depending on the goal. If the goal is to find packaged retroviruses, then the enrichment should protect them. If the goal is to find activity within the cell, the lab work may involve stranded RNA-seq. This work still needs to be developed, but they are preparing the pipeline to support it. The counter-screening step can potentially remove some of the endogenous retroviruses, for example, by identifying those situations where the read matches the retrovirus and the host equally well. However, the approach still needs to be developed to ensure retrovirus safety. **Song Sun** added that they have been testing PhyloID for retrovirus signal detection. For endogenous retroviruses, the signal obtained with and without benzonase treatment does not differ.

**Siemon Ng** asked a question about validation in modules and how it's impacted by going from short-to long-read approaches or for transcriptomics? **Tom de Man** answered that the K-mer approach is independent of the read length, and it can be used beyond Illumina. **Robert Charlebois** clarified that the PhyloID pipeline is adapted for short reads, but not all modules would be impacted by long reads. The adjustment could be performed specifically for affected modules. **Vanessa Sarathy** agreed that this would be part of the risk assessment, and some adjustments would be needed to adapt to long reads.

A question was directed to the panel asking whether different K-mer sizes had been tested. **Robert Charlebois** answered that they tested K-mers of lengths 4,5 and 6, and they all performed equally well. They finally settled on 6 because these were faster. After a follow-up question regarding the use of K-mer frequency instead of identity, he clarified that short reads do not have enough information for frequency-based K-mer counting. K-mer counting is used in phylogenomics because the entire genomes contain sufficient information to generate accurate distance matrices. **Tom de Man** answered that because they work with nucleotides, they use K-mers of length 35, which is the maximum allowed. He was further asked how to handle cases where the reference sequence is not in the database. Do they risk missing samples with ≈95 % identity because the K-mer is too long? He clarified that sequences not matching the viral or cell-line database correspond to a very low proportion of reads. They may consider including those reads in the qualification step for the read mapping analysis, but he foresees that the difference would be small given the diversity in the database.

**Robert Charlebois** was asked if the NGS tests for bacterial detection could replace some of the current tests or only be used as a follow-up for identification. He further commented that *Mycoplasma* would be a good test for entering this space because it is a long assay. Dr. Charlebois answered that sterility testing would not likely be replaced because it is a straightforward test. However, he agreed that *Mycoplasma* could be detected via nucleic acid tests, but they could miss some species if they are too specific. Thus, NGS would solve that. Bacteria present an opposite problem to viruses in that their sequences tend to be very conserved. Thus, they would likely only be identified to the genus level. The sensitivity of bacterial detection would increase the challenge concerning the background signal.

A final comment addressed future challenges that may arise from personalized medicine therapies. **Vanessa Sarathy** replied that at the moment, these platforms and approaches are used for a limited number of applications. This would require a different mindset and feasibility assessments. **Tom de Man** added that they use the human genome sequence from the Telomere-to-Telomere consortium to remove the human background in HeLa cell line analyses. Using the actual cell line genome would remove HPV18, which has to be detected.

## 6 Session 5: implementation of NGS for biologics

The final session was a panel discussion moderated by **Ivana Knezevic**, **Laurent Mallet**, and **Arifa Khan**. The panel was composed of participants representing different expertise in NGS (including technical, bioinformatics, and regulatory) from diverse organizations and different global regions.

• **Johannes Blümel**, PEI, Germany
• **Ken Kono**, National Institute of Health Sciences, Japan
• **Marc Eloit**, Pathoquest, France
• **Alison Armstrong**, Merck KGaA, Germany
• **Carine Logvinoff**, Sanofi, France
• **Blandine de Saint-Vis**, Boehringer Ingelheim Health Animal, France
• **Marie Murphy**, Eli Lilly & Co, Ireland
• **Siemon Ng**, Notch Therapeutics, Canada
• **Christophe Lambert**, GSK, Belgium
• **Gwenaël Cirefice**, EDQM, France
• **Pei-Ju Chin**, U.S. FDA, U.S.A.
• **Ajmeer Ramkishan**, Ministry of Health & Family Welfare, India (Online)
• **Koji Ishii**, National Institute of Infectious Diseases, Japan (Online)

**Ivana Knezevic** opened the session by introducing the panelists attending online and inviting them to present the current situation in their countries in terms of implementing NGS for the evaluation of biologics.

**Ajmeer Ramkishan** described the situation in India where many (vaccine) manufacturers have been WHO-prequalified. He explained that India promotes the use of NGS methods, having developed well-defined guidelines for the use of NGS for the evaluation of biological products. These guidelines are part of the good manufacturing practices published as per the provisions of the Drugs and Cosmetic Act provisions and by the Indian Council of Medical Research in 2017. The Indian Government also published the New Drugs and Clinical Trials Rules in 2019 (G S R 227E), which foresees the use of NGS as an alternative method for adventitious agent detection. India also follows the WHO TRS and ICH guidelines as part of the efforts to ensure viral vaccine safety. However, India lacks the expertise to support the validation of these methods, as well as cell lines and other raw materials, and would benefit from capacity building and a well-defined protocol for validation. Vaccine manufacturers should take the lead. The Indian Pharmacopoeia needs to incorporate the use of NGS, and regulators are preparing to adopt NGS as an alternative method to *in vivo* testing. India benefits from the support of the and NIBSC U.K. and, as an IABS member, aims to collaborate in efforts to harmonize methods for viral safety and promote global health.

**Koji Ishii** described the situation in Japan where the NRAs have not received any NGS submissions. The regulatory framework in Japan is complex, with the evaluation of different types of biologics dispersed through different NRAs. Several live vaccines have been produced by small domestic companies in Japan over the last 50 years. These manufacturers evaluated viral safety using *in vivo* and *in vitro* cell culture tests, and they are reluctant to replace these methods with NGS. Dr. Ishii noted that given the limitations of these methods presented in this conference, it is important to encourage these domestic companies to introduce these new sequencing methods. Ken Kono agreed that Japan is conservative concerning the adoption of NGS for product evaluation, but the revision of the ICH Q5A guideline is raising the interest of the NRAs in this new technology.

**Ivana Knezevic** commented that Japan was a founding member of ICH, but its conservative position shows the importance of developing manufacturers' expertise and regulatory preparedness. New technologies are usually first implemented by industry, but regulators need time to build knowledge and experience to evaluate them. IABS events can help build this trust, and countries’ Pharmacopoeias can help define guidelines. Dr. Knezevic further encouraged the panel to discuss the most important steps to move forward with implementation.

**Laurent Mallet** commented that although the ICH Q5A (R2) [1] applies mainly to biotherapeutic products and the scope has now been expanded to include some viral vaccines and gene therapy products that can undergo viral clearance, its principles apply to all biological products. The new Ph. Eur. chapter 2.6.41 has also been drafted for applying NGS to all biological products, including viral vaccines and gene therapy products [12]. Thus, there are no product-specific barriers to the applicability of NGS methods. As with previous chapters of the Ph. Eur. (e.g., Ph. Eur. 2.6.7 [58]) that have boosted the use of new technologies, the new chapter on NGS with validation guidelines [12] is also expected to advance implementation.

**Johannes Blümel** added that regulators have difficulties assessing new methodologies that they do not fully understand. Dissemination, training, and refence materials are essential for promoting acceptance. It is also important to stay flexible because the technology is rapidly evolving.

**Arifa Khan** noted the importance of the various virus spiking studies presented at the conference and others that are ongoing or being initiated in the AVDTWG, as well as additional external efforts. These studies are a step toward developing general SOPs for optimization of NGS for virus detection in different types of matrices relevant to biological test materials, which are needed for broader NGS implementation.

**Marie Murphy** added that the introduction of NGS is a significant component of the overall revised ICH Q5A(R2) guideline. Since its publication, the Implementation Working Group has been developing training materials for key guideline updates: prior knowledge, continuous manufacturing, the introduction of new product types, for example genetically engineered viral vectors and viral vector-derived products. The application of NGS has been integrated throughout the case studies. These include direct examples of new product types and conventional monoclonal antibody production platforms where NGS can be used as a replacement or supplementary method for the conventional assays. The ICH will disseminate these training materials in 2025.

**Ivana Knezevic** noted that expertise building takes time and is particularly challenging for countries that are starting 15 or 20 years behind other regions of the world. The priority targets for capacity building are manufacturing countries where regulators are exposed to local manufacturers as well as those from foreign countries. Training materials from previous discussions, meeting reports, and online webinars contain much information already available. However, all this information can be overwhelming for those only starting and should be summarized into simpler educational tools.

**Arifa Khan** highlighted the importance of the AVDTWG discussions as a training source. These working group and subgroup meetings are opportunities for interactive exchanges with scientific experts and for knowledge sharing. She invited non-members interested in learning more about NGS to join the AVDTWG for their meetings, which are held every two months.

**Alison Armstrong** noted the role of IABS conferences in bringing together regulators and manufacturers. Although regulators have not yet generally accepted these methods, they are on a journey to acceptance. In her experience, one-on-one meetings with regulatory authorities are beneficial in providing training and advice. Practical sessions may be necessary to support technical discussions. When regulators and manufacturers are familiar with the technology, its challenges, and its potential, then there will be more support for implementation. Simplified and summarized training materials, such as those being prepared by the ICH, will also be useful.

**Ivana Knezevic** introduced the topic of biologicals of assured quality. Regulatory authorities play a key role in assuring the quality, safety, and efficacy of medical products. Effective regulatory systems are an essential component of health systems and contribute to desired public health outcomes and innovation. The Global Benchmarking Tool (GBT) represents the primary means by which the WHO objectively evaluates regulatory systems, as mandated by WHA Resolution 67.20 on Regulatory System Strengthening for medical products [59]. The GBT also incorporates the concept of ‘maturity level’ or ML (adapted from ISO 9004 [60]), allowing WHO and regulatory authorities to assess the overall ‘maturity’ of the regulatory system on a scale of 1 (existence of some elements of regulatory system) to 4 (operating at advanced level of performance and continuous improvement). In that context, the role of regulators is very important. An early dialogue between regulators and manufacturers before submission may save time, accelerate the review of the data, and contribute to building the regulators' expertise in these topics.

**Marc Eloit** commented on challenges related to reference standards. The WHO reference virus panel has been successfully and widely used in viromics and genomics analyses. However, reference cell lines for transcriptomics are lacking. Such standards would be useful for comparing different pipelines. **Arifa Khan** noted that they are initiating a study to evaluate virus detection from infected cell lines with different levels of expression, which were developed in her laboratory. In this study, the virus panel is also added to mimic a complex matrix in a background of unprocessed bulk. Based on the results, a collaborative study will be opened to evaluate the suitability of using the cell lines as reference material across different laboratories’ protocols and bioinformatics pipelines.

Since the audience had expressed difficulties in accessing the WHO reference virus panel, this topic was further discussed to gain more insight into the nature of these challenges. An important consideration was international shipping. The documents requested by customs may vary from one country to another, and with different timelines. All documents must be reviewed and approved by the courier before shipment. In Belgium, virus stocks need to be shipped with a permit via a specific EU airport for inspection, designated in the permit. Otherwise, they are destroyed or returned to the sender. Problems occurred with couriers shipping via other airports. It would help to have the stocks shipped from a central location in Europe. **Ivana Knezevic** added that WHO reference materials must be stored/shipped via WHO custodian laboratories. For this purpose, she suggested MHRA in the UK, which currently distributes 99 % of all WHO standards all over the world. However, the audience expressed concerns with the documentation needed for the UK and proposed an EU country instead. Furthermore, this would not solve the issues with shipments to other regions of the world. The regulations also vary depending on the virus’ stability and active versus inactive status. There have been general issues with shipping virus-infected cells and animal-derived material as well. **Heather Couch**, an American Type Culture Collection (ATCC) representative, added that ATCC ships different kinds of cells to different countries by preparing CITES (the Convention on International Trade in Endangered Species of Wild Fauna and Flora) permits for each country beforehand. Clear communication can help understand the needs of each country to allow the preparation of all documentation ahead of shipment. Ivana Knezevic concluded this point by stating that these issues must be considered when preparing future standards and that a first step should be to create at least one more distribution point. This will not solve all the issues, but it will reduce them for many countries.

Addressing a question from the audience, **Ivana Knezevic** explained that the WHO reliance model has brought benefits across different areas, particularly for vaccines. It allowed for the establishment of regulatory networks that collaborate to assess new products and make decisions. For example, in a network established in Africa, regulatory authorities who are more advanced in evaluating a product, share their experience with other countries. This helps in gaining experience, building trust and relying on each other's regulatory decisions. These networks also benefit from the support of external regulators, such as or CBER. This process is sometimes slow because not all regulatory authorities are willing to engage in the reliance concept fully, and some prefer to keep their independent assessment. This also happens with WHO prequalification. In this case, the WHO provides the evaluation for regulatory authorities without sufficient expertise, but the final decision is made by the authority. Although the regulatory authorities generally accept the prequalification assessment, in some cases, they prefer to conduct their independent review. The reliance model encompasses the full product assessment and not a specific methodology (e.g. NGS). However, the WHO aims to understand the regulators' expectations via the survey and other discussions to clarify how this reliance model could be further explored for producing countries and for other countries. **Laurent Mallet** explained that EDQM coordinates a network of batch-release laboratories in Europe where testing by one National Control Laboratory is mutually recognized by all the other laboratories from the network. This was particularly successful during the COVID-19 pandemic when there was a need for a quick procedure to test and release vaccine batches. This experience has shown the difficulty of having a laboratory equipped with all the technologies and the advantage of specialized centers for different technologies. It may also be important for NGS to be implemented via specialized centers in different regions. Regulators need some level of expertise to evaluate the dossier, but they do not all need to have hands-on experience in the technology.

A short discussion followed on the use of the term NGS versus HTS. This question had been addressed previously during the development of the ICH Q5A (R2) and the new Ph.Eur. chapter. Overall, it was recognized that both terms can be used interchangeably, and both documents specify that both terms refer to the same technology.

**Siemon Ng** commented on cell therapy. Many regulatory bodies have separate groups for cell and gene therapy and vaccines, and their views on the use of NGS do not always align. The ICH Q5A (R2) [1] and the Ph.Eur. chapter 2.6.41 [12] are helpful, but it is still challenging to apply the use of NGS in the Cell and Gene Therapy field. **Ivana Knezevic** noted that the Ph. Eur. chapter 2.6.41 is being elaborated to cover cell therapy products, and that the WHO has also received questions on how to evaluate them. Cell and gene therapy products require special attention in different aspects, particularly as the material is precious and limited. These should be considered in future discussions.

**Marc Eloit** added that one point pending clarification is the expected LOD. Some agencies are requesting LODs that are not reachable even by PCR, which may be a challenge during the evaluation stage. **Arifa Khan** noted that the situation is evolving, and different Offices are trying to align on these aspects. This evolution is driven by the submissions with the data and from spiking studies, which aim to establish harmonized protocols. Ultimately, it may be possible to reach a realistic LOD threshold. As the technology evolves, this may need to be adapted, and regulators may suggest improving the LOD when they have seen evidence that it can be improved. **Laurent Mallet** noted that, though it is important to progress on the sensitivity, regulatory documents did not specify a target for the conventional assays. It is not crucial to be more stringent with NGS than with previously used assays because the breadth of detection with NGS is much greater than the other assays. Furthermore, **Arifa Khan** added that the LOD varies with the test material, and full testing package needs to be evaluated. Thus, for now the LOD is assessed case by case.

**Carine Logvinoff** asked what would make regulators accept a transcriptomics approach as a substitution for a release test, considering that currently, there are no reference standards. Arifa Khan noted that their experience with transcriptomics in regulatory submissions is limited, but other studies, such as the study presented by Marc Eloit and the AVDTWG spiking study #3 to assess transcriptomics [2], have been valuable in presenting LODs. These can be considered when evaluating transcriptomics submissions at this time. In these cases, working with the sponsor to agree on an acceptable LOD is important. Generally, there are still no formal recommendations for NGS, but the work has been developed hand in hand between regulators and sponsors. The transcriptomics approach has advantages related to the abundance of transcripts. However, the ongoing studies are optimized for detection because all cells are infected. In a cell substrate, if only a few cells are infected, transcripts might not be detectable. **Marc Eloit** agreed that there are two expression levels to evaluate: the ratio of infected to non-infected cells and the number of RNAs per cell. However, it is not clear which is the most important. The detection is aimed to be as sensitive as possible, and ultimately this needs to be evaluated case by case. **Johannes Blümel** suggested a collaborative study comparing the genomics and transcriptomics approaches on an infected cell culture.

A comment from the audience highlighted the importance of the breadth of detection. It is critical to implement the technology because it allows to detect more contamination cases and not be held back on details regarding LOD thresholds. For cell and gene therapy products, even if the method has not been fully demonstrated, it is important to use NGS because it will allow the detection of more contamination events. **Arifa Khan** clarified that the aim was never to discourage the use of NGS; however, there is a learning curve in which regulators and manufacturers need to understand the technology and discuss how to use it best. **Johannes Blümel** added that the field of ATMP is different in the type of primary materials used, but it would benefit from the breadth of detection in NGS. **Carine Logvinoff** noted that here, the assessment should be done on the general performance of the assay and not on the sensitivity for a particular virus. **Arifa Khan** summarized that NGS is undoubtedly the superior assay in terms of the breadth of detection and that its sensitivity is affected by the matrix. This must be considered in terms of suitability for the intended purpose.

On this point, the audience commented that the current validations might be overfitting the pipelines to model viruses and neglecting the breadth of detection. **Arifa Khan** clarified that though the aim is to demonstrate the detection of all viruses, it is important to evaluate the differences between viruses and test the characteristics of different virus families. **Johannes Blümel** added that there may always be gaps, but the model viruses have been carefully selected to represent a breadth of viral characteristics. Although each new method and source material requires reevaluation, the WHO virus panel also allows for confidence in the wet lab validation against novel viruses. Challenging the pipelines with various synthetic sequences could help fill the gap in bioinformatic detection. **Gibran Rubio Quintanares** noted that ML further increases the risk of biasing systems by this limited panel of selected viruses. The topic of standards for bioinformatic analysis will be discussed further in the AVDTWG subgroup DE meetings.

The last question to the panelists was their most important take-home messages on NGS implementation.

• **Gwenaël Cirefice** indicated the call to improve regulatory acceptance and wider implementation of NGS beyond Europe and North America, and the need to support both manufacturers working on a GMP environment and regulators. These points will be considered as the comments to the new Ph.Eur. chapter 2.6.41 on HTS are being addressed.
• **Johannes Blümel** noted the need for expertise in handling large, metagenomic data and to improve access to reference viruses.
• **Christophe Lambert** highlighted the progress in NGS technology over the past years and its increasing adoption worldwide despite some challenges.
• **Ken Kono** noted that it is time to implement NGS technology and stressed the importance of international discussions and multi-center spiking studies.
• **Carine Logvinoff** appreciated the opportunity to have the innovation space within a company that values exploring the technology before implementation. She further highlighted the need to strengthen the team as developing this technology requires expertise.
• **Ivana Knezevic** acknowledged all the participation and achievements shared in the conference. She noted that much work is still needed to ensure NGS can be used as a regulatory tool at a global level. This includes building expertise, sharing knowledge, and ensuring accessibility. She further highlighted the practical issues regarding the use of the WHO virus panel and the need to facilitate its broader availability and use.
• **Siemon Ng** agreed with Ivana Knezevic and noted the tremendous collaborative work being done to reach the current point and the importance of the current guidelines for new applications such as cell and gene therapy.
• **Pei-Ju Chin** also highlighted the advances achieved so far. He was encouraged by the fact that a general agreement on the acceptable range of sensitivities could be achieved even without a standardized protocol. He further stressed that CBER/FDA is available to meet with the manufacturers before they implement NGS technologies.
• **Marc Eloit** highlighted the acceleration in understanding and acceptance of NGS, which might replace conventional tests more rapidly than anticipated. He further stressed the need to upscale bioinformatics capabilities.
• **Blandine de Saint-Vis** noted the progress since the last meeting and the increasing community interest in implementing NGS. She highlighted the challenges faced in the veterinary field but stressed the progress in database curation, particularly the potential of using retroviruses, which may allow for new applications for avian cells.
• **Arifa Khan** shared that NGS implementation has been a process. Acknowledging how far this process has come is encouraging, but it is also burdensome to recognize that some challenges persist. The openness in the conference presentations and discussions stresses the need to continue encouraging this type of initiative. These conferences and publications also provide a resource for those who have not started with NGS. The collaborative effort of regulators, CROs, and sponsors has been crucial to reach the current point. Many questions remain unanswered, but they are being solved as the technology evolves.
• **Laurent Mallet** was impressed with the journey already achieved. He recognized the need to quickly finalize the Ph.Eur. chapter 2.6.41 on HTS, as this is expected to boost implementation.

### 6.1 Conclusions

**Laurent Mallet** stated the conference was successful in meeting the goals and provided an opportunity for networking and exchanging experiences among the experts and with other participants. A good consensus was achieved on the status of NGS implementation. NGS was agreed to be superior to classical methods in terms of breadth of detection and overall quality and performance of the technology, particularly for replacing the *in vivo* assays. The latest data on scientific developments and regulatory submissions was shared, with specific examples of industry applications using NGS. Some examples of NGS applications beyond cell-based biological products (e.g. blood/plasma) were also presented. Important discussions were held on the expectations and requirements for method validation. A specific LOD target was not defined, as the need for flexibility in the implementation at this time was recognized. Joining the AVDTWG was highlighted as important for learning and advancing the field of NGS for AV detection. The IABS board will discuss the plans for a future NGS meeting. **Laurent Mallet** closed the meeting by acknowledging all contributors, participants, and organizers.

## CRediT authorship contribution statement

**Arifa S. Khan:** Writing – original draft, Writing – review & editing, Conceptualization, Supervision. **Laurent Mallet:** Writing – original draft, Writing – review & editing, Conceptualization. **Johannes Blümel:** Writing – review & editing. **Noémie Deneyer:** Writing – review & editing. **Sigrid De C.J. Keersmaecker:** Writing – review & editing. **Blandine de Saint-Vis:** Writing – review & editing. **Ivana Knezevic:** Writing – review & editing. **Carine Logvinoff:** Writing – review & editing. **Marie Murphy:** Writing – review & editing. **Siemon H.S. Ng:** Writing – review & editing. **Yoji Sato:** Writing – review & editing. **Michael Wall:** Writing – review & editing. **Ana Goios:** Writing – original draft, Writing – review & editing, Supervision. **Pieter Neels:** Project administration, Writing – review & editing.

## Disclaimer

This article is the work product of an employee or group of employees of government and other organizations. However, the statements, opinions, or conclusions contained therein do not necessarily represent statements, opinions, or conclusions of any government agency or organization. The use of commercial product names is specified for experimental procedures only and does not constitute recommendation or endorsement by any of the authors, organizations, or agencies. The presentations and discussions included in this meeting report are the views of the individual speakers and panelists and do not necessarily represent those of the authors or their organizations.

## Declaration of generative AI and AI-assisted technologies in the writing process

During the preparation of this work, meeting transcripts were generated using Fireflies.ai (https://fireflies.ai), and text editing was supported by Grammarly (https://www.grammarly.com). The speakers and panelists reviewed and edited the content and are responsible for the content of the publication.

## Declaration of competing interest

Laurent Mallet and Siemon H.S. Ng hold shares in Sanofi. Carine Logvinoff is an employee of Sanofi and holds shares in Sanofi. Noémie Deneyer is an employee of the GSK group of companies and holds shares in GSK. Ana Goios is an employee and holds shares in P95. All other authors have no financial conflict of interest.
